# Comparative efficacy of long-acting bronchodilators for COPD - a network meta-analysis

**DOI:** 10.1186/1465-9921-14-100

**Published:** 2013-10-07

**Authors:** Shannon Cope, James F Donohue, Jeroen P Jansen, Matthias Kraemer, Gorana Capkun-Niggli, Michael Baldwin, Felicity Buckley, Alexandra Ellis, Paul Jones

**Affiliations:** 1 MAPI Consultancy, Toronto, Canada; 2 Department of Medicine, University North Carolina, North Carolina, USA; 3 MAPI Consultancy, Boston, USA; 4 Novartis Pharma AG, Basel, Switzerland; 5 Novartis Horsham Research Centre, Horsham, UK; 6 Division of Clinical Science, St George’s University of London, London SW17 0RE, UK

**Keywords:** COPD, Bronchodilator, Systematic review, Meta-analysis, Mixed treatment comparison

## Abstract

**Background:**

Clinicians are faced with an increasingly difficult choice regarding the optimal bronchodilator for patients with chronic obstructive pulmonary disease (COPD) given the number of new treatments. The objective of this study is to evaluate the comparative efficacy of indacaterol 75/150/300 μg once daily (OD), glycopyrronium bromide 50 μg OD, tiotropium bromide 18 μg/5 μg OD, salmeterol 50 μg twice daily (BID), formoterol 12 μg BID, and placebo for moderate to severe COPD.

**Methods:**

Forty randomized controlled trials were combined in a Bayesian network meta-analysis. Outcomes of interest were trough and post-dose forced expiratory volume in 1 second (FEV_1_), St. George’s Respiratory Questionnaire (SGRQ) score and responders (≥4 points), and Transition Dyspnea Index (TDI) score and responders (≥1 point) at 6 months.

**Results:**

Indacaterol was associated with a higher trough FEV_1_ than other active treatments (difference for indacaterol 150 μg and 300 μg versus placebo: 152 mL (95% credible interval (CrI): 126, 179); 160 mL (95% CrI: 133, 187)) and the greatest improvement in SGRQ score (difference for indacaterol 150 μg and 300 μg versus placebo: -3.9 (95% CrI -5.2, -2.6); -3.6 (95% CrI -4.8, -2.3)). Glycopyrronium and tiotropium 18 μg resulted in the next best estimates for both outcomes with minor differences (difference for glycopyrronium versus tiotropium for trough FEV_1_ and SGRQ: 18 mL (95% CrI: -16, 51); -0.55 (95% CrI: -2.04, 0.92).

**Conclusion:**

In terms of trough FEV_1_ and SGRQ score indacaterol, glycopyrronium, and tiotropium are expected to be the most effective bronchodilators.

## Background

Patients with chronic obstructive pulmonary disease (COPD) experience airway obstruction, involving reduced lung function and health-related quality of life due to symptoms such as breathlessness and exacerbations [1]. Since COPD is a progressive disease, the main objective of treatment is to improve lung function, prevent and control symptoms, and ultimately to improve health status. Bronchodilator medications are central to symptom management in COPD, with long-acting preparations preferred over short-acting ones [1].

The classes of inhaled long-acting bronchodilators available for COPD are long-acting β2-agonists (LABAs) (i.e. indacaterol 75 μg, 150 μg or 300 once daily (OD), salmeterol 50 μg twice daily (BID), or formoterol 12 μg (BID) and long-acting muscarinic antagonists (LAMAs, also called anticholinergic bronchodilators) (i.e. tiotropium bromide 18 μg or 5 μg OD). Recently two more LAMAs, aclidinium bromide 400 mg BID and glycopyrronium bromide 44 μg OD were recently approved by the European Medicines Agency and the Food and Drug Administration.

Given the number of the alternative long-acting treatments available for COPD, clinicians are faced with an increasingly challenging choice regarding the optimal treatment. Since there is no head-to-head randomized controlled trial (RCT) that evaluates all the different monotherapies available, and it is unlikely that such a trial will ever be performed (given the increasing number of options available), a comprehensive systematic review and network meta-analysis is of interest to synthesize the RCT evidence. The objective of the current analysis was to evaluate the comparative efficacy of long-acting bronchodilators in patients with moderate to severe COPD in terms of lung function, health status, and dyspnoea. Approved bronchodilators or those for which data was available at the time of the literature search were included: indacaterol 75/150/300 μg OD, salmeterol 50 μg BID, formoterol 12 μg BID, tiotropium bromide 18 μg or 5 μg OD, aclidinium bromide 200 μg OD and glycopyrronium bromide 50 μg OD. No evidence for the approved dose of aclidinium bromide was available at the time of the search, therefore results for aclidinium bromide 200 μg OD were included in the analysis but are not presented given that this dose has not been approved.

## Methodology

### Identification and selection of articles

A systematic literature search was performed to identify RCTs evaluating the efficacy of the long-acting monotherapies for COPD. MEDLINE® and EMBASE® databases were searched simultaneously for the period of 1989 to July 2011 and the Cochrane Library was also searched. Search terms included a combination of free-text and thesaurus terms relevant to COPD, the treatments of interest, and RCTs (see Additional file 1). Cope et al. 2012 searched the literature from 1989–2010 and this was updated for 2010–2011 for the treatments of interest. The relevance of each citation identified was based on title and abstract (or full-text article) according to predefined selection criteria:

*Population:* Adults with COPD;

*Interventions:* Indacaterol 75/150/300 μg OD, tiotropium 5/18 μg OD, salmeterol 50 μg BID, formoterol 12 μg BID, aclidinium 200/400 μg OD, and glycopyrronium 50 μg OD;

*Comparators*: Any of the interventions evaluated as monotherapy or placebo;

*Outcomes*: Trough forced expiratory volume in 1 second (FEV_1_), post-dose FEV_1_ (2 hours after dosing), St. George’s Respiratory Questionnaire (SGRQ) total score and proportion of patients with an improvement of at least 4 units in SGRQ total score (“SGRQ Responders” [2]), Transition Dyspnoea Index (TDI) total score and proportion of patients with an improvement of at least 1 unit in TDI score (“TDI Responders” [3]), proportion of patients with an exacerbation and exacerbation rate;

*Study Design*: RCTs.

In addition to the studies identified in the systematic review, clinical trial reports were provided by Novartis for trials evaluating indacaterol and glycopyrronium, all of which had been published at the time of the search except for the trial B2333 (NCT00792805) [4-14].

### Outcomes of interest

The outcomes of interest included trough FEV_1_, post-dose FEV_1_, SGRQ total score, SGRQ responders, TDI total score, and TDI responders. Change from baseline was evaluated for all continuous outcomes, with the exception of TDI which was evaluated at follow-up. The current analysis focuses on results at 6 months (discussed in the following), although endpoints were also analyzed at 12 weeks (see online Additional file 1). Exacerbation outcomes will be evaluated in separate manuscript in order to account for differences in definitions.

### Data extraction

Information related to the study and patient characteristics was extracted for the included studies, which allowed for a comprehensive assessment of the similarities and differences across the trials. For each outcome the mean results and the associated uncertainty (i.e. standard error) were extracted where sufficient information was available within a two week range for each time point of interest (i.e. between 22–26 weeks for 6 month time point). If necessary, the software DigitizIt version 1.5.8 was used to extract data from graphs presented in the publications.

### Network meta-analysis

Bayesian network meta-analysis (NMA) models were used [15-18] to simultaneously synthesize the results of the included studies for each outcome of interest.

NMAs within the Bayesian framework involve data, a likelihood distribution, a model with parameters, and prior distributions [19]. The model relates the data from the individual studies to basic parameters reflecting the (pooled) treatment effect of each intervention relative to placebo as the overall reference treatment. Based on these basic parameters, the relative efficacy between each of the interventions was obtained. For the continuous outcomes a normal likelihood distribution was used and for the binary outcomes a binomial likelihood was used [16,17,20,21]. For each analysis, both fixed and random effects models were evaluated. With a NMA, randomization only holds within a trial and not across trials. Consequently, there is the risk that patients who were studied in different comparisons are not similar, which may lead to consistency violations. In order to minimize confounding bias, analyses with a constant treatment by covariate interaction were evaluated [22] or analyses were performed excluding specific trials to assess the impact of potential treatment effect modifiers. Potential treatment effect modifiers were identified a priori as concomitant treatments, COPD severity, smoking status, age, and sex. Separate analyses were performed to evaluate the potential treatment effect modifiers given the limited number of studies included in each analysis. Non-informative prior distributions for the model parameters were implemented to avoid influencing the results of the analysis based on the prior beliefs.

For each model with and without covariates, both fixed and random effects models were tested. The deviance information criterion was used to compare the models, which provides a measure of model fit that penalizes model complexity accordingly [23]. The random effects model was selected unless there was sufficient evidence to suggest the fit of the fixed effect model was better. The analyses were performed using WinBUGS 1.4.1 statistical software [24].

**Figure 1:**
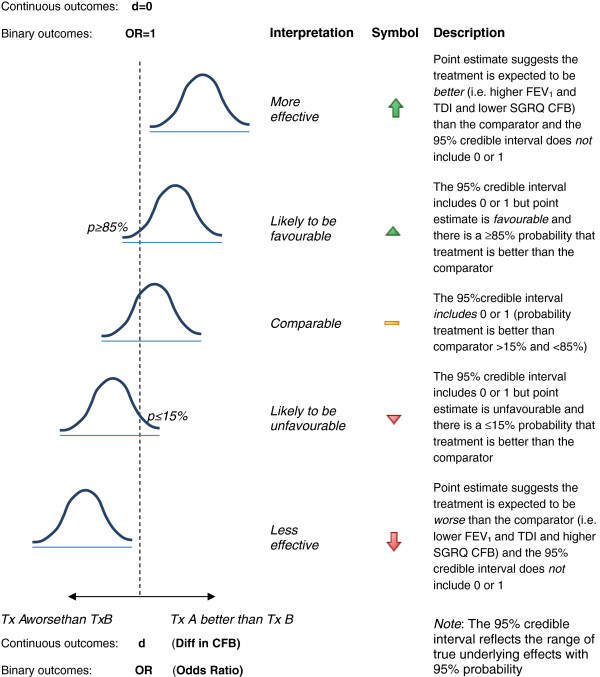
**Interpretation of the relative treatment effects resulting from the network meta-analysis for continuous and binary outcomes.** CFB=Change from baseline; D=difference in CFB; FEV_1_=Forced expiratory volume; OR=odds ratio; SGRQ=St. George’s Respiratory Questionnaire; TDI=Transitional Dyspnoea Index; d=0 indicates that the dotted line is equal to a difference in CFB between the treatments of zero for continuous endpoints (i.e. no difference between treatments); OR=1 indicates that the dotted line is equal to an odds ratio of one for binary endpoints (i.e. no difference between treatments).

The results of the NMA are presented in terms of ‘point estimates’ for the relative treatment effects and the 95% credible intervals (95% CrI). The probability that each treatment is *best* is also presented which is calculated based on the proportion of Markov chain Monte Carlo cycles in which a specific treatment ranks first out of the total (where the ranking is based on the treatment effect size) [25]. Figure 1 outlines the interpretation of the results for continuous and binary outcomes, which utilizes the probability that one treatment is *better* than another (i.e. proportion of cycles in which specific treatment estimate is better than the comparator).

## Results

### Evidence base

**Figure 2:**
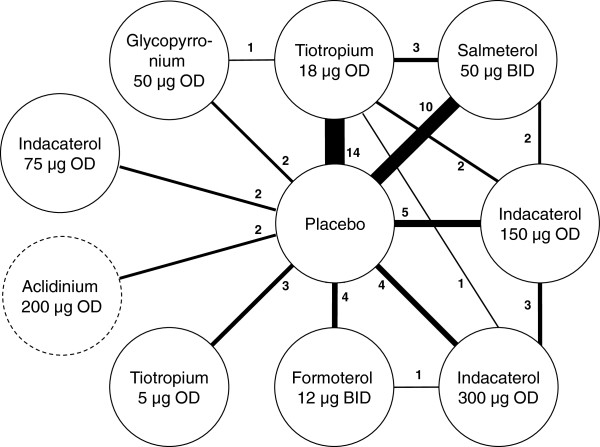
**Network of RCTs included in the network meta-analysis.** Note: The thickness of the lines corresponds to the number of RCTs available for each treatment comparison; Two RCTs comparing aclidinium 200 μg OD to placebo were included in the network of evidence but results were not presented since this dose has not been approved.

The systematic review identified 51 RCTs, of which 40 RCTs were included in the NMA (See Additional file 1: Figure S1 in the online supplement) [4-14,26-52]. Eleven RCTs that did not report the outcomes of interest within the time frames of interest were excluded [53-63] (see flow chart in Additional file 1: Figure S1). Figure 2 illustrates the network of RCTs included in the NMA and the key study characteristics are presented in the online supplement (see Additional file 1: Table S1).

All studies were parallel placebo-controlled multi-center RCTs, with the exception of three RCTs that compared active treatments only [5,13,28]. All trials were double-blind, although three RCTs evaluated open-label tiotropium [7,11,51]. The RCTs were generally considered of good quality, but the method of randomization and concealment of treatment allocation were not always well reported.

The study designs were mostly similar with some differences in terms of the study location and background medications. The studies were predominantly European and North American, although two trials were based in Asia [4,12] and some trials included study centers in South America, Africa, and Asia [6,7,14]. Most RCTs allowed patients to receive a concomitant ICS, whereas some RCTs allowed the continued use of LABAs [26,35,37,44,45,48] or LAMAs [30,36].

**Figure 3:**
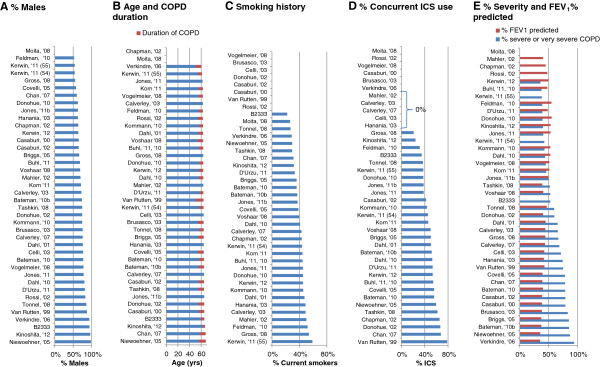
**Patient characteristics of the randomized controlled trials used for the network meta-analysis.** Note: Zero values indicate not reported unless otherwise indicated; Figure B presents the mean duration of COPD in years (red) per study and the mean age per study (blue+ red).

The enrolled patients had a COPD diagnosis, were 40 years of age or older and were current or ex-smokers with a smoking history of at least 10 years. Selected RCTs included patients with a smoking history of at least 15 years [34] or 20 years [6,7,9,12,14,41,43]. Generally, patients were required to have an FEV_1_/ FVC of less than or equal to 0.70 and an FEV_1_ percent predicted often between 30 and 80%, although this range varied across the studies (see Additional file 1: Table S1). Exacerbation history was reported in only five of the RCTs [8,11,31,36,42], and two studies specified inclusion criteria with respect to exacerbations history, requiring at least one exacerbation over the prior one to two years [35] or one exacerbation per year over the prior three years [30]. Figure 3 illustrates the variation in the RCTs in terms of the proportion of males (range: 52-99%), average age (range: 60–68 years), duration of COPD (range: 3.8-13.1 years), proportion of current smokers (range: 22-59%), proportion receiving ICS during the trial (range: 0-78%), and the proportion with severe or very severe COPD (range: 36-95%) as reported based on the GOLD criteria or calculated as function of the FEV_1_% predicted. Overall, differences were most apparent in terms of ICS use and severity.

### Network meta-analysis

**Figure 4:**
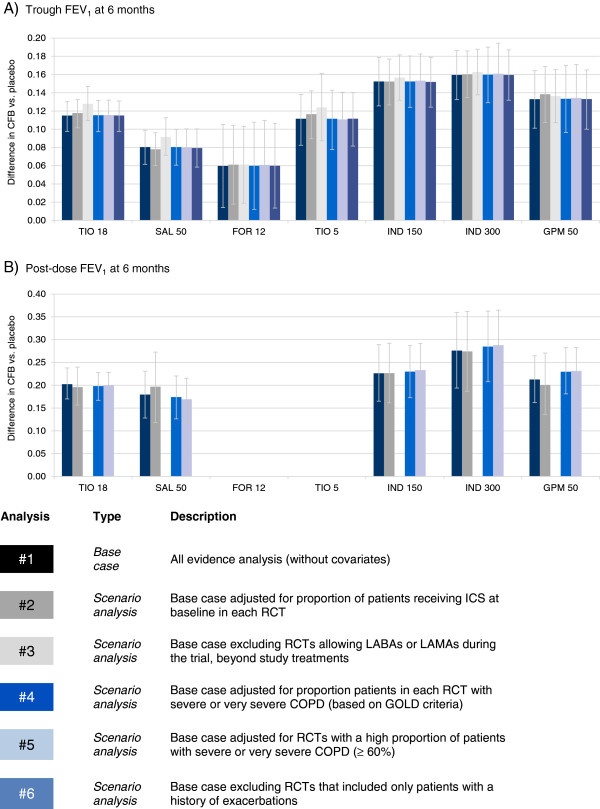
**Trough and post-dose FEV**_**1 **_**network meta-analysis results at 6 months: Difference in change from baseline (CFB) versus placebo.** Bars represent 95% Credible Interval; CFB = Change from baseline; FEV_1_ = Forced expiratory volume; FOR 12 = Formoterol 12 μg twice daily (BID); GPM 50 = Glycopyrronium 50 μg once daily (OD); IND 150 = Indacaterol 150 μg OD; IND 300 = Indacaterol 300 μg OD; SAL 50 = Salmeterol 50 μg BID; TIO 5 = Tiotropium 5 μg OD; TIO 18 = Tiotropium 18 μg OD.

**Figure 5:**
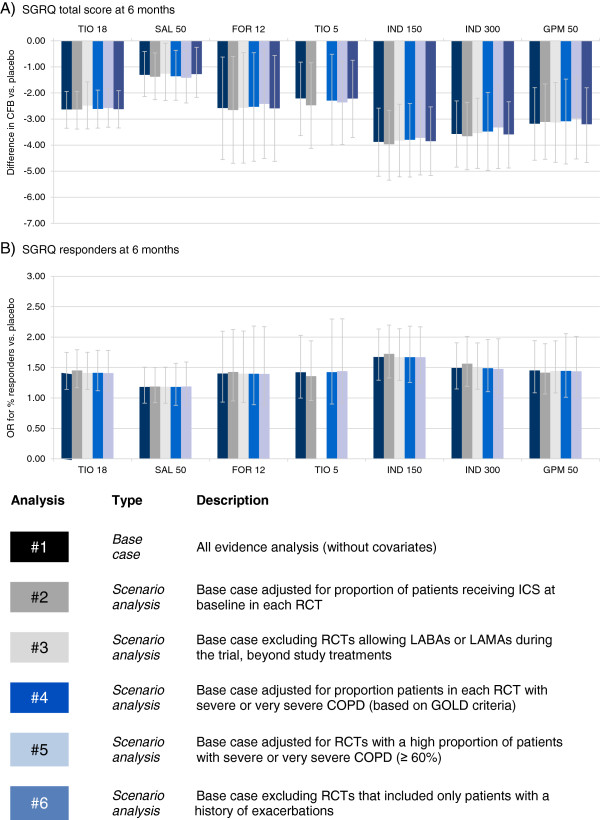
**SGRQ network meta-analysis results at 6 months: Difference in change from baseline (CFB) or odds ratio (OR) for responders versus placebo.** Bars represent 95% Credible Interval; CFB = Change from baseline; FOR 12 = Formoterol 12 μg twice daily (BID); GPM 50 = Glycopyrronium 50 μg once daily (OD); IND 150 = Indacaterol 150 μg OD; IND 300 = Indacaterol 300 μg OD; OR = Odds ratio; SAL 50 = Salmeterol 50 μg BID; SGRQ = St. George’s Respiratory Questionnaire; TIO 5 = Tiotropium 5 μg OD; TIO 18 = Tiotropium 18 μg OD.

**Figure 6:**
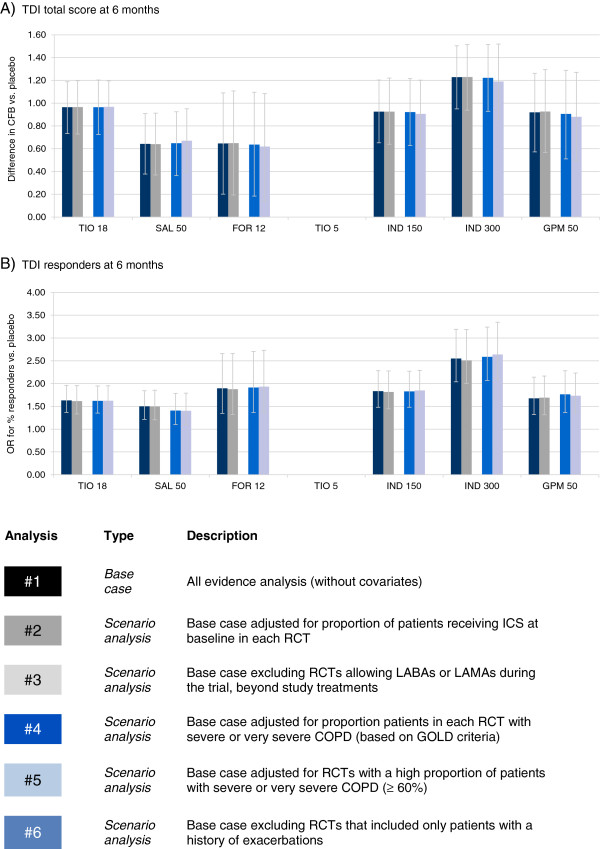
**TDI network meta-analysis results at 6 months: Difference in change from baseline (CFB) or odds ratio (OR) for responders versus placebo.** Bars represent 95% Credible Interval; CFB = Change from baseline; FOR 12 = Formoterol 12 μg twice daily (BID); GPM 50 = Glycopyrronium 50 μg once daily (OD); IND 150 = Indacaterol 150 μg OD; IND 300 = Indacaterol 300 μg OD; OR = Odds ratio; SAL 50 = Salmeterol 50 μg BID; TDI = Transition Dyspnoea Index; TIO 5 = Tiotropium 5 μg OD; TIO 18 = Tiotropium 18 μg OD.

The RCTs were synthesized with a network meta-analysis. The individual study results are presented in an online supplement (See Additional file 1: Tables S2-7). In the base case analysis all RCTs were included without covariates. Scenario analyses were performed to explore the impact of differences identified in terms of concomitant ICS use, concomitant LABAs or LAMAs, COPD severity, and exacerbation history that were considered most likely to cause bias. These covariates were selected based on the extent of the variation across the RCTs and any evidence regarding treatment effect modifiers in the individual studies. Initially the results for the base case analysis at 6 months are presented by outcome. Figures 4, 5, and 6 present the base case and scenario analyses at 6 months, which illustrate the results of the NMA for each treatment versus placebo in terms of lung function, health status, and dyspnoea, respectively. The last section summarizes the impact of the scenario analyses across the outcomes.

#### Trough and post-dose forced expiratory volume in 1 second

In terms of change from baseline (CFB) in lung function, results from the base case analysis suggest that there is 64% probability that indacaterol 300 μg provides the greatest improvement in trough FEV_1_ and an 83% probability of the greatest effect in post-dose FEV_1_. Indacaterol 150 μg (29%; 10%) and glycopyrronium (6%; 6%) are less likely to provide the greatest improvement in these outcomes out of all interventions compared. (The probability of each treatment being the best is presented in online supplement (see Additional file 1: Table S8)). Tables 1 and 2 present the treatment effect estimates for each treatment comparison for trough and post-dose FEV_1_, respectively. In terms of trough FEV_1_ all treatments are expected to be more efficacious than placebo. The largest difference in trough FEV_1_ was between indacaterol and formoterol (range in point estimates for difference in CFB for indacaterol 150-300 μg: 93-100 mL), although indacaterol was also more efficacious than salmeterol 50 μg (72-79 mL), tiotropium 5 μg (41-48 mL) and tiotropium 18 μg (37-44 mL). Both glycopyrronium 50 μg and tiotropium 18 μg were more efficacious than formoterol 12 μg (73 mL and 55 mL, respectively) and salmeterol 50 μg (53 mL and 35 mL, respectively) in terms of trough FEV_1_. The availability of data for post-dose FEV_1_ was more limited; although the treatments were all more efficacious than placebo no differences were detected between the active treatments.

**Table 1:** **Results of base case NMA: Difference in intervention versus the comparator for CFB in trough FEV**_**1 **_**(mL) at 6 months, 95% credible intervals, and probability that the intervention is better than the comparator**

**Intervention**		**Comparator**															
		**PLACEBO**		**TIO 18**		**SAL 50**		**FOR 12**		**TIO 5**		**IND 150**		**IND 300**		**GPM 50**	
PLACEBO	estimate	0		−115		−80		−60		−111		−152		−160		−133	
	95% CrI			−132	−100	−99	−62	−105	−14	−140	−85	−179	−126	−187	−133	−165	−102
	P(better)			<1%		<1%		1%		<1%		<1%		<1%		<1%	
TIO 18	estimate	115		0		35		55		3		−37		−44		−18	
	95% CrI	100	132			12	58	8	104	−29	36	−66	−8	−74	−14	−51	16
	P(better)	>99%				>99%		99%		59%		1%		<1%		14%	
SAL 50	estimate	80		−35		0		21		−31		−72		−79		−53	
	95% CrI	62	99	−58	−12			−28	70	−65	2	−102	−42	−111	−47	−89	−17
	P(better)	>99%		0%				81%		3%		<1%		<1%		<1%	
FOR 12	estimate	60		−55		−21		0		−52		−93		−100		−73	
	95% CrI	14	105	−104	−8	−70	28			−106	1	−143	−42	−145	−54	−129	−18
	P(better)	99%		1%		19%				3%		<1%		<1%		1%	
TIO 5	estimate	111		−3		31		52		0		−41		−48		−21	
	95% CrI	85	140	−36	29	−2	65	−1	106			−79	−2	−86	−9	−63	21
	P(better)	>99%		41%		97%		97%				2%		1%		15%	
IND 150	estimate	152		37		72		93		41		0		−7		19	
	95% CrI	126	179	8	66	42	102	42	143	2	79			−39	25	−21	60
	P(better)	>99%		99%		>99%		>99%		98%				32%		83%	
IND 300	estimate	160		44		79		100		48		7		0		27	
	95% CrI	133	187	14	74	47	111	54	145	9	86	−25	39			−15	67
	P(better)	>99%		>99%		>99%		>99%		99%		68%				90%	
GPM 50	estimate	133		18		53		73		21		−19		−27		0	
	95% CrI	102	165	−16	51	17	89	18	129	−21	63	−60	21	−67	15		
	P(better)	>99%		86%		>99%		99%		85%		17%		10%			

*95% CrI* = 95% Credible Interval; *P(better)* = Probability of being the treatment being better than the comparator in terms of the outcome assessed; *CFB* = Change from baseline; *FEV*_*1*_ = Forced expiratory volume; *FOR 12* = Formoterol 12 μg twice daily (BID); *GPM 50* = Glycopyrronium 50 μg once daily (OD); *IND 150* = Indacaterol 150 μg OD; *IND 300* = Indacaterol 300 μg OD; *SAL 50* = Salmeterol 50 μg BID; *TIO 5* = Tiotropium 5 μg OD; *TIO 18* = Tiotropium 18 μg OD.

**Table 2:** **Results of base case NMA: Difference in intervention versus the comparator for CFB in post-dose FEV**_**1 **_**(mL) at 6 months, 95% credible intervals, and probability that the intervention is better than the comparator**

**Intervention**		**Comparator**											
		**PLACEBO**		**TIO 18**		**SAL 50**		**IND 150**		**IND 300**		**GPM 50**	
PLACEBO	estimate	0		−202		−180		−226		−276		−213	
	95% CrI			−235	−167	−231	−128	−287	−163	−358	−192	−263	−160
	P(better)			<1%		<1%		<1%		<1%		<1%	
TIO 18	estimate	202		0		23		−24		−0.074		−10	
	95% CrI	167	235			−39	82	−91	42	−158	10	−67	45
	P(better)	>99%				78%		22%		4%		33%	
SAL 50	estimate	180		−23		0		−46		−96		−33	
	95% CrI	128	231	−82	39			−117	26	−190	−1	−104	40
	P(better)	>99%		22%				9%		2%		16%	
IND 150	estimate	226		24		46		0		−50		14	
	95% CrI	163	287	−42	91	−26	117			−140	40	−66	92
	P(better)	>99%		78%		91%				13%		65%	
IND 300	estimate	276		74		96		50		0		64	
	95% CrI	192	358	−10	158	1	190	−40	140			−33	159
	P(better)	>99%		96%		98%		87%				91%	
GPM 50	estimate	213		10		33		−14		−64		0	
	95% CrI	160	263	−45	67	−40	104	−92	66	−159	33		
	P(better)	>99%		67%		84%		35%		9%			

*95% CrI* = 95% Credible Interval; *P(better)* = Probability of being the treatment being better than the comparator in terms of the outcome assessed; *CFB* = Change from baseline; *FEV*_*1*_ = Forced expiratory volume; *FOR 12* = Formoterol 12 μg twice daily (BID); *GPM 50* = Glycopyrronium 50 μg once daily (OD); *IND 150* = Indacaterol 150 μg OD; *IND 300* = Indacaterol 300 μg OD; *SAL 50* = Salmeterol 50 μg BID; *TIO 5* = Tiotropium 5 μg OD; *TIO 18* = Tiotropium 18 μg OD.

#### St. George’s Respiratory questionnaire

In terms of health status, base case results indicated there is a ~50% probability that indacaterol 150 μg is the most efficacious in terms of SGRQ total score and SGRQ responders, which was followed by indacaterol 300 μg (25%; 10%), formoterol 12 μg (6%; 13%), and glycopyrronium (15%; 12%). Tables 3 and 4 present the treatment effect estimates for each treatment comparison for SGRQ total score and responders, respectively. All active treatments are expected to be more efficacious than placebo for SGRQ total score. For SGRQ response only indacaterol 150 μg and 300 μg, tiotropium 18 μg and glycopyrronium 50 μg were more efficacious than placebo, whereas the credible intervals for the other treatment estimates versus placebo include 1. With respect to SGRQ total score, indacaterol was more efficacious than salmeterol 50 μg (indacaterol 150 μg/300 μg difference point estimates ranging from −2.26 to −2.56 points), as was glycopyrronium 50 μg (−1.87 points) and tiotropium 18 μg (−1.32 points), although an improved response was only observed for the comparison of indacaterol 150 μg versus salmeterol (odds ratio (OR) of 1.42).

**Table 3:** Results of base case NMA: Difference in intervention versus the comparator for CFB in SGRQ total score at 6 months, 95% credible intervals, and probability that the intervention is better than the comparator

**Intervention**		**Comparator**															
		**PLACEBO**		**TIO 18**		**SAL 50**		**FOR 12**		**TIO 5**		**IND 150**		**IND 300**		**GPM 50**	
PLACEBO	estimate	0.00		2.63		1.31		2.58		2.21		3.87		3.57		3.18	
	95% CrI			1.91	3.31	0.48	2.21	0.60	4.53	0.78	3.61	2.56	5.17	2.30	4.84	1.78	4.56
	P(better)			<1%		<1%		1%		<1%		<1%		<1%		<1%	
TIO 18	estimate	−2.63		0.00		−1.32		−0.05		−0.42		1.24		0.94		0.55	
	95% CrI	−3.31	−1.91			−2.32	−0.21	−2.09	2.01	−1.98	1.17	−0.15	2.63	−0.41	2.31	−0.92	2.04
	P(better)	>99%				99%		52%		72%		4%		8%		22%	
SAL 50	estimate	−1.31		1.32		0.00		1.27		0.91		2.56		2.26		1.87	
	95% CrI	−2.21	−0.48	0.21	2.32			−0.92	3.36	−0.82	2.50	1.03	4.00	0.74	3.75	0.21	3.44
	P(better)	>99%		1%				12%		13%		<1%		<1%		1%	
FOR 12	estimate	−2.58		0.05		−1.27		0.00		−0.37		1.30		1.00		0.61	
	95% CrI	−4.53	−0.60	−2.01	2.09	−3.36	0.92			−2.78	2.05	−0.97	3.56	−0.96	2.98	−1.81	3.02
	P(better)	99%		48%		88%				62%		13%		16%		31%	
TIO 5	estimate	−2.21		0.42		−0.91		0.37		0.00		1.67		1.36		0.97	
	95% CrI	−3.61	−0.78	−1.17	1.98	−2.50	0.82	−2.05	2.78			−0.29	3.58	−0.53	3.26	−1.01	2.94
	P(better)	>99%		28%		87%		38%				4%		7%		15%	
IND 150	estimate	−3.87		−1.24		−2.56		−1.30		−1.67		0.00		−0.30		−0.70	
	95% CrI	−5.17	−2.56	−2.63	0.15	−4.00	−1.03	−3.56	0.97	−3.58	0.29			−1.82	1.24	−2.59	1.28
	P(better)	>99%		96%		>99%		87%		96%				65%		77%	
IND 300	estimate	−3.57		−0.94		−2.26		−1.00		−1.36		0.30		0.00		−0.39	
	95% CrI	−4.84	−2.30	−2.31	0.41	−3.75	−0.74	−2.98	0.96	−3.26	0.53	−1.24	1.82			−2.28	1.47
	P(better)	>99%		92%		>99%		84%		93%		35%				66%	
GPM 50	estimate	−3.18		−0.55		−1.87		−0.61		−0.97		0.70		0.39		0.00	
	95% CrI	−4.56	−1.78	−2.04	0.92	−3.44	−0.21	−3.02	1.81	−2.94	1.01	−1.28	2.59	−1.47	2.28		
	P(better)	>99%		78%		99%		69%		85%		23%		34%			

*95% CrI* = 95% Credible Interval; *P(better)* = Probability of being the treatment being better than the comparator in terms of the outcome assessed; *CFB* = Change from baseline; *FOR 12* = Formoterol 12 μg twice daily (BID); *GPM 50* = Glycopyrronium 50 μg once daily (OD); *IND 150* = Indacaterol 150 μg OD; *IND 300* = Indacaterol 300 μg OD; *SAL 50* = Salmeterol 50 μg BID; *SGRQ* = St. George’s Respiratory Questionnaire; *TIO 5* = Tiotropium 5 μg OD; *TIO 18* = Tiotropium 18 μg OD.

**Table 4:** Results of base case NMA: Difference in intervention versus the comparator for SGRQ responders at 6 months in terms of odds ratios (ORs), 95% credible intervals, and probability that the intervention is better than the comparator

		**Comparator**															
**Intervention**		**PLACEBO**		**TIO 18**		**SAL 50**		**FOR 12**		**TIO 5**		**IND 150**		**IND 300**		**GPM 50**	
PLACEBO	estimate	1.00		0.71		0.84		0.71		0.70		0.60		0.67		0.69	
	95% CrI			0.61	0.83	0.70	1.00	0.48	1.07	0.49	1.00	0.47	0.77	0.52	0.87	0.52	0.92
	P(better)			<1%		9%		5%		3%		<1%		<1%		1%	
TIO 18	estimate	1.41		1.00		1.20		1.01		0.99		0.84		0.95		0.97	
	95% CrI	1.21	1.64			0.92	1.58	0.65	1.58	0.66	1.50	0.64	1.14	0.71	1.29	0.71	1.35
	P(better)	>99%				92%		52%		48%		11%		35%		43%	
SAL 50	estimate	1.19		0.84		1.00		0.84		0.83		0.71		0.79		0.81	
	95% CrI	1.00	1.42	0.63	1.09			0.53	1.34	0.54	1.27	0.52	0.96	0.57	1.11	0.56	1.17
	P(better)	91%		8%				22%		15%		2%		8%		12%	
FOR 12	estimate	1.40		0.99		1.18		1.00		0.98		0.84		0.94		0.96	
	95% CrI	0.93	2.10	0.63	1.55	0.74	1.90			0.58	1.68	0.53	1.33	0.63	1.42	0.59	1.58
	P(better)	95%		48%		78%				47%		20%		36%		44%	
TIO 5	estimate	1.42		1.01		1.20		1.02		1.00		0.85		0.95		0.98	
	95% CrI	1.00	2.03	0.67	1.52	0.79	1.87	0.60	1.74			0.56	1.32	0.63	1.49	0.62	1.55
	P(better)	97%		52%		85%		53%				19%		39%		46%	
IND 150	estimate	1.67		1.19		1.42		1.20		1.18		1.00		1.12		1.15	
	95% CrI	1.29	2.13	0.88	1.57	1.04	1.92	0.75	1.88	0.76	1.79			0.83	1.51	0.78	1.67
	P(better)	>99%		89%		98%		80%		81%				79%		79%	
IND 300	estimate	1.49		1.06		1.26		1.07		1.05		0.89		1.00		1.03	
	95% CrI	1.15	1.91	0.78	1.41	0.90	1.76	0.71	1.59	0.67	1.60	0.66	1.20			0.70	1.49
	P(better)	>99%		65%		92%		64%		61%		21%				56%	
GPM 50	estimate	1.45		1.03		1.23		1.04		1.02		0.87		0.97		1.00	
	95% CrI	1.08	1.94	0.74	1.42	0.85	1.79	0.63	1.71	0.65	1.61	0.60	1.27	0.67	1.43		
	P(better)	99%		57%		88%		56%		54%		21%		44%			

*95% CrI* = 95% Credible Interval; *P(better)* = Probability of being the treatment being better than the comparator in terms of the outcome assessed; *FOR 12* = Formoterol 12 μg twice daily (BID); *GPM 50* = Glycopyrronium 50 μg once daily (OD); *IND 150* = Indacaterol 150 μg OD; *IND 300* = Indacaterol 300 μg OD; *OR* = Odds ratio; *SAL 50* = Salmeterol 50 μg BID; *SGRQ* = St. George’s Respiratory Questionnaire; *TIO 5* = Tiotropium 5 μg OD; *TIO 18* = Tiotropium 18 μg OD.

#### Transition dyspnoea index

For TDI, base case results suggest there is an 86% probability that indacaterol 300 μg is the best treatment as measured with the total score; In a responder analysis using TDI, a 95% probability was obtained with indacaterol 300 μg, which was followed by formoterol 12 μg (4%), and indacaterol 150 μg (1%), and glycopyrronium (1%). Tables 5 and 6 present the treatment effect estimates for each treatment comparison for TDI total score and responders, respectively. Indacaterol 300 μg and tiotropium were more efficacious than salmeterol 50 μg in terms of total score (difference of 0.58 and 0.32, respectively) and indacaterol 300 μg was also more efficacious than formoterol 12 μg (difference of 0.58). A greater response was observed with indacaterol 300 μg in comparison to salmeterol 50 μg (OR of 1.70), tiotropium 18 μg (OR of 1.56), glycopyrronium 50 μg (OR = 1.52) and indacaterol 150 μg (OR of 1.39).

**Table 5:** Results of base case NMA: Difference in intervention versus the comparator for TDI total score at 6 months, 95% credible intervals, and probability that the intervention is better than the comparator

**Intervention**		**Comparator**													
		**PLACEBO**		**TIO 18**		**SAL 50**		**FOR 12**		**IND 150**		**IND 300**		**GPM 50**	
PLACEBO	estimate	0.00		−0.96		−0.64		−0.65		−0.93		−1.23		−0.92	
	95% CrI			−1.20	−0.74	−0.91	−0.38	−1.09	−0.20	−1.20	−0.65	−1.51	−0.95	−1.27	−0.58
	P(better)			<1%		<1%		<1%		<1%		<1%		<1%	
TIO 18	estimate	0.96		0.000		0.32		0.32		0.04		−0.27		0.046	
	95% CrI	0.741	1.195			0.012	0.64	−0.17	0.81	−0.28	0.37	−0.59	0.07	−0.33	0.43
	P(better)	>99%				98%		90%		59%		6%		60%	
SAL 50	estimate	0.64		−0.32		0.00		0.00		−0.29		−0.58		−0.28	
	95% CrI	0.38	0.91	−0.64	−0.01			−0.52	0.51	−0.63	0.06	−0.96	−0.22	−0.71	0.14
	P(better)	>99%		2%				49%		5%		<1%		10%	
FOR 12	estimate	0.65		−0.32		0.00		0.00		−0.28		−0.58		−0.27	
	95% CrI	0.20	1.09	−0.81	0.17	−0.51	0.52			−0.77	0.22	−1.02	−0.14	−0.83	0.29
	P(better)	>99%		10%		51%				13%		1%		17%	
IND 150	estimate	0.93		−0.04		0.29		0.28		0.00		−0.30		0.01	
	95% CrI	0.65	1.20	−0.37	0.28	−0.06	0.63	−0.22	0.77			−0.63	0.02	−0.43	0.45
	P(better)	>99%		41%		95%		87%				3%		51%	
IND 300	estimate	1.23		0.27		0.58		0.58		0.30		0.00		0.31	
	95% CrI	0.95	1.51	−0.07	0.59	0.22	0.96	0.14	1.02	−0.02	0.63			−0.13	0.75
	P(better)	>99%		94%		>99%		99%		97%				92%	
GPM 50	estimate	0.92		−0.05		0.28		0.27		−0.01		−0.31		0.00	
	95% CrI	0.58	1.27	−0.43	0.33	−0.14	0.71	−0.29	0.83	−0.45	0.43	−0.75	0.13		
	P(better)	>99%		40%		90%		83%		49%		8%			

*95% CrI* = 95% Credible Interval; *P(better)* = Probability of being the treatment being better than the comparator in terms of the outcome assessed; *FOR 12* = Formoterol 12 μg twice daily (BID); *GPM 50* = Glycopyrronium 50 μg once daily (OD); *IND 150* = Indacaterol 150 μg OD; *IND 300* = Indacaterol 300 μg OD; *SAL 50* = Salmeterol 50 μg BID; *TIO 5* = Tiotropium 5 μg OD; *TIO 18* = Tiotropium 18 μg OD; *TDI* = Transitional Dyspnoea Index.

**Table 6:** Results of base case NMA: Difference in intervention versus the comparator for TDI responders at 6 months in terms of odds ratios (ORs), 95% credible intervals, and probability that the intervention is better than the comparator

		**Comparator**													
**Intervention**		**PLACEBO**		**TIO 18**		**SAL 50**		**FOR 12**		**IND 150**		**IND 300**		**GPM 50**	
PLACEBO	estimate	1.00		0.61		0.67		0.53		0.55		0.39		0.60	
	95% CrI			0.51	0.73	0.54	0.82	0.38	0.74	0.44	0.68	0.31	0.49	0.47	0.76
	P(better)			<1%		<1%		<1%		<1%		<1%		<1%	
TIO 18	estimate	1.63		1.00		1.09		0.86		0.89		0.64		0.97	
	95% CrI	1.37	1.96			0.88	1.37	0.59	1.26	0.70	1.14	0.50	0.83	0.75	1.27
	P(better)	>99%				78%		22%		17%		<1%		42%	
SAL 50	estimate	1.50		0.92		1.00		0.79		0.82		0.59		0.89	
	95% CrI	1.21	1.84	0.73	1.14			0.54	1.17	0.63	1.06	0.44	0.78	0.66	1.21
	P(better)	>99%		22%				11%		6%		<1%		23%	
FOR 12	estimate	1.90		1.16		1.26		1.00		1.03		0.74		1.13	
	95% CrI	1.35	2.66	0.80	1.68	0.85	1.87			0.70	1.52	0.53	1.04	0.74	1.71
	P(better)	>99%		78%		89%				56%		4%		72%	
IND 150	estimate	1.83		1.12		1.22		0.97		1.00		0.72		1.09	
	95% CrI	1.48	2.28	0.88	1.44	0.95	1.58	0.66	1.43			0.55	0.94	0.80	1.50
	P(better)	>99%		83%		94%		44%				1%		72%	
IND 300	estimate	2.55		1.56		1.70		1.35		1.39		1.00		1.52	
	95% CrI	2.04	3.19	1.21	2.02	1.28	2.28	0.97	1.89	1.07	1.81			1.10	2.10
	P(better)	>99%		>99%		>99%		96%		99%				99%	
GPM 50	estimate	1.68		1.03		1.12		0.89		0.92		0.66		1.00	
	95% CrI	1.32	2.14	0.79	1.34	0.82	1.52	0.58	1.35	0.67	1.26	0.48	0.91		
	P(better)	>99%		58%		77%		28%		28%		1%			

*95% CrI* = 95% Credible Interval; *P(better)* = Probability of being the treatment being better than the comparator in terms of the outcome assessed; *FOR 12* = Formoterol 12 μg twice daily (BID); *GPM 50* = Glycopyrronium 50 μg once daily (OD); *IND 150* = Indacaterol 150 μg OD; *IND 300* = Indacaterol 300 μg OD; *OR* = Odds ratio; *SAL 50* = Salmeterol 50 μg BID; *TIO 5* = Tiotropium 5 μg OD; *TIO 18* = Tiotropium 18 μg OD; *TDI* = Transitional Dyspnoea Index.

#### Scenario analyses

**Figure 7:**
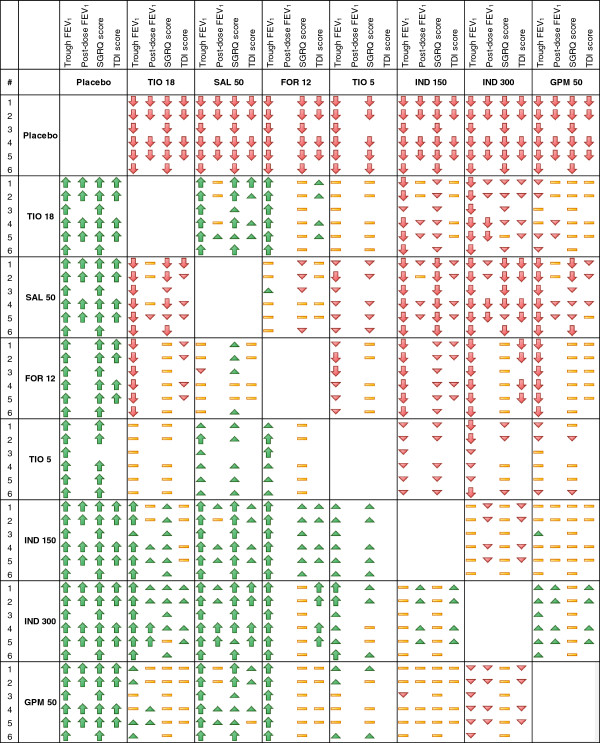
**Overview of the network meta-analysis results for trough FEV**_**1**_**, post-dose FEV**_**1**_**, SGRQ total score, and TDI total score at 6 months.** Symbols are explained in Figure 1; FEV_1_ = Forced expiratory volume in 1 second; FOR 12 = Formoterol 12 μg twice daily (BID); GPM 50 = Glycopyrronium 50 μg once daily (OD); IND 150 = Indacaterol 150 μg OD; IND 300 = Indacaterol 300 μg OD; SAL 50 = Salmeterol 50 μg BID; SGRQ = St. George’s Respiratory Questionnaire; TDI = Transition Dyspnoea Index; TIO 5 = Tiotropium 5 μg OD; TIO 18 = Tiotropium 18 μg OD.

An overview of the NMA results for the continuous outcomes at 6 months is presented in Figure 7 for the different scenarios using symbols to summarize the main conclusion for each comparison and analysis. Treatment effect estimates were most sensitive to adjustment for disease severity (i.e. degree of airflow limitation). Results were less sensitive to adjustment for concomitant ICS use or concomitant LABA or LAMA use. Minimal changes were observed by excluding studies that required an exacerbation history. Overall, the different meta-regression analyses resulted in minimal changes in the treatment effect estimates and did not alter the interpretation.

## Discussion

The objective of this analysis was to compare the efficacy of individual bronchodilators for patients with moderate to severe COPD in terms of lung function, health status, and dyspnoea. Based on the results of the NMA at 6 months, indacaterol resulted in the best treatment at either the 150 or 300 μg dose, depending on the outcome assessed, although indacaterol was not always more efficacious than the alternative bronchodilators and differences versus other active treatments were small.

Thresholds for clinically important differences have been established for active treatments versus placebo in terms of FEV_1_, SGRQ total score, and TDI total score. Although comparisons of alternative active treatments may not be expected to reach these thresholds, in the absence of any clear guidance for interpretation of active treatments these thresholds have been used to help identify whether differences between treatments are clinically relevant. FEV_1_ thresholds defined to evaluate whether an active treatment has demonstrated a clinically meaning difference versus placebo (i.e. 100 mL-140 mL [64-66]) suggest that none of the differences in lung function between the active treatments were clinically meaningful in the analysis of all studies without covariates (base-case analysis). In terms of health status, improvements in SGRQ total score were identified for indacaterol 150 and 300 μg, glycopyrronium 50 μg, and tiotropium 18 μg in comparison to salmeterol, although differences were less than the 4 units for a clinically relevant difference. Only indacaterol 150 μg led to a clinically relevant response relative to salmeterol with respect to SGRQ. The estimated differences between treatments in terms of TDI total score were smaller than the threshold for clinically relevant difference of 1 point. A greater proportion of patients had a clinically relevant TDI response with indacaterol 300 μg in comparison to salmeterol 50 μg and tiotropium 18 μg.

The validity of the findings depends on the quality of the RCTs and the extent of any violations in the similarity and consistency assumptions across studies [22]. In a network meta-analysis of RCTs involving multiple treatment comparisons, the randomization holds only within the individual trials, and not across trials. If the different direct comparisons show systematic differences in study and patient characteristics, and these differences are treatment effect modifiers, then the estimates of any indirect comparison as obtained with the network meta-analysis will be biased. With a meta-regression analysis we aim to minimize this confounding bias by adjusting for inconsistencies in the evidence base.

The trials included in the network meta-analysis were generally of good quality. All trials were blinded with the exception of open label tiotropium 18 μg in three RCTs, which has been shown to be comparable to blinded results for FEV_1_, although with some minimal bias introduced on more subjective measures [67]. However, some differences across trials were identified in terms of concomitant ICS use, concomitant LABA or LAMA use, the severity of COPD and the exacerbation history requirements. Individual scenario analyses were performed to evaluate these differences using a either a meta-regression model or by excluding specific studies that differed in terms of the characteristics identified. Overall, the interpretation of findings obtained with analysis based on all studies without adjustment for covariates (base-case analysis) was the same as obtained with the scenario analyses in the majority of cases. Only a few scenarios suggest a slight difference in the strength of the comparative effects.

Although we went to great lengths to assess whether the network meta-analysis was biased by systematic differences between studies, the meta-regression analysis was based on study level data which has limitations. First of all, it was not feasible to include all covariates of interest simultaneously due to the limited number of data points. Second, study and patient characteristics were not consistently reported. For example, limited information was available for the exacerbation history of patients and therefore it was only possible to exclude trials that clearly required an exacerbation history. Similarly, information regarding COPD comorbidities was not consistently reported across the RCTs, so these potential differences could not be explored. Third, it is well known that meta-regression analysis based on study level data can be prone to ecological bias, which means that association between study level patient characteristics and the treatment effects may not reflect the individual-level effect modification of that covariate. As such, it has to be accepted that there is the risk of residual confounding bias.

This study aimed to provide a comprehensive evidence base. However, it was not possible to capture all recent studies. The literature search did not capture the ACCORD [68] or ATTAIN [69] trials evaluating aclidinium 400 μg BID that were published after the date of the search, and an updated analysis including these studies is of interest. Moreover, data for new bronchodilators that may be of interest, such as vilanterol, olodaterol, and unmeclidinium, were not available and may necessitate an updated analysis including these treatments in future. It should also be noted, that in order to include the indacaterol and glycopyrronium trials that were not yet published at the time of the literature search, Novartis provided the corresponding clinical study reports. No attempt was made to obtain study reports from manufacturers of formoterol, salmeterol, or tiotropium. This may have induced a bias, but it is unlikely that key positive results were withheld from the primary papers.

The current paper focussed on the 6 month time point, but results at 12 weeks are available as well (online Additional file 1). Results at 6 months provide better insight regarding efficacy over a longer term than the 12 week results, particularly for patient reported outcomes, but data for the approved dose of indacaterol in the United States (75 μg) are only available at 12 weeks. There is also a need to evaluate whether there is sufficient data available to inform decision-makers regarding longer term comparative efficacy. One potential limitation of the current analysis is that some studies were excluded from the analysis if the outcomes reported deviated by more than 2 weeks from the specified time points of interest. For example, the analyses of SGRQ and trough FEV_1_ at the 6 months excluded data from Stockley et al. 2006 (n = 634), and Chan et al. 2007 (n = 913), which may have influenced the results somewhat for salmeterol and tiotropium. However, given the large number of studies included for these treatments, the exclusion of these trials is not expected to have a large impact. Similarly, only post-dose FEV_1_ results at 2 hours after dosing were included, which reflected the most commonly reported time point.

Although several network meta-analyses have been published in the area of COPD, it is challenging to compare the current results to previous analyses. Earlier analyses did not include indacaterol [70,71], and more recent analyses have focussed on comparisons to fixed-dose combinations [72,73] or were not as comprehensive in terms of the data or outcomes evaluated. For example, the analysis by Cope et al. 2012 [74] was restricted to four trials from the indacaterol trial program and the review by Cope et al. 2012 [75] focussed on trough FEV_1_ and SGRQ total score at 12 weeks. Furthermore, these studies did not include evidence regarding treatments such as glycopyrronium 50 μg or tiotropium 5 μg. Recent meta-analyses restricted the evidence to RCTs that directly compared the active interventions of interest [76,77] or placebo controlled trials [78] without considering the full network of evidence. Also, in some cases alternative LABAs were pooled together (i.e. formoterol, salmeterol, and/or indacaterol), despite potential differences in these treatments, preventing a clear comparison to the current results. Therefore, to our knowledge, the current study generates new evidence regarding the efficacy of monotherapies for moderate to severe COPD.

The efficacy outcomes analyzed here provide insight into a broad range of clinically relevant outcomes. FEV_1_ is often a primary endpoint and reflects an important outcome from a clinical and regulatory perspective, providing a reproducible and objective measurement of airflow limitation [1]. SGRQ and TDI are based on validated instruments and provide unique insight into the patient perspective. Exacerbations reflect another key outcome given their impact on quality of life and resource utilization, although a separate publication will be developed in order to capture the complexity associated with these outcomes. It should be acknowledged that no safety outcomes were assessed, which is a critical aspect of decision-making that has been addressed by others [79-83].

In conclusion, based on the results of the NMA, indacaterol, glycopyrronium, and tiotropium are expected to be the most favourable bronchodilators in terms of lung function, health status, and dyspnoea at six months, although differences were only clinically meaningful for indacaterol 150 μg in comparison to salmeterol in terms of SGRQ and for indacaterol 300 μg in comparison to salmeterol, tiotropium and formoterol in terms of TDI response.

## Competing interests

SC, JJ, FB, and AE are employees of Mapi and received funding from Novartis for the study. JD and PJ received no compensation for working on this manuscript. JD served as a consultant for Novartis and received and honorarium in the area of drug development. PJ has received honorarium for speaker fees and/or advisory boards from Almirall, AstraZeneca, Bayer, GlaxoSmithKline, Merck, Novartis, Roche, Spiration. St. George’s University of London has received consulting fees for work that PJ has performed for Almirall, GlaxoSmithKline, and Novartis. MK was a full time employee of Novartis Pharma AG until February 2013. GC is a full time employee of Novartis Pharma AG and has shares in the company. MB is a full time employee of Novartis UK and does not have shares in the company.

## Authors’ contributions

All authors participated in the development of this manuscript. SC, JJ, FB, AE participated in all stages of the study design, systematic literature view, statistical analyses and manuscript development. JD, MK, GC, MB, PJ participated in the study design and coordination and helped to draft the manuscript. All authors read and approved the final manuscript.

## Supplementary Material

Additional file 1 **RCT study and patient characteristics, individual study results, flow diagram, and NMA results at 12 weeks. Table S1.** Key study characteristics for RCTs included in the network meta-analysis. **Table S2.** Individual study results for trough FEV_1_ at 12 weeks and 6 months (mL): difference in change from baseline (CFB) for treatment versus comparator. **Table S3.** Individual study results for post-dose FEV_1_ at 12 weeks and 6 months (mL): difference in change from baseline (CFB) for treatment versus comparator. **Table S4.** Individual study results for SGRQ total score at 12 weeks and 6 months: difference in change from baseline (CFB) for treatment versus comparator. **Table S5.** Individual study results for SGRQ responders at 12 weeks and 6 months: n/N (proportion responders) for each treatment. **Table S6.** Individual study results for TDI total score at 12 weeks and 6 months: difference in change from baseline (CFB) for treatment versus comparator. **Table S7.** Individual study results for TDI responders at 12 weeks and 6 months: n/N (proportion responders) for each treatment. **Table S8.** Results of base case network meta-analysis: Probability of each treatment being the best in terms of trough and post-dose FEV_1_ (mL), SGRQ total score and response, and TDI total score and response at 6 months. **Figure S1.** Flow diagram of study selection. **Figure S2.** Trough and post-dose FEV_1_ network meta-analysis results at 12 weeks: Difference in change from baseline (CFB) versus placebo. **Figure S3.** SGRQ total score network meta-analysis results at 6 months: Difference in change from baseline (CFB) or odds ratio (OR) versus placebo. **Figure S4.** TDI total score network meta-analysis results at 6 months: Difference in change from baseline (CFB) or odds ratio (OR) versus placebo. Click here for file
