# Supplementary material for: Comparative efficacy of long-acting bronchodilators for COPD - a network meta-analysis
Source: Respir Res. 2013 Oct 7;14(1):100. doi: 10.1186/1465-9921-14-100 (PMC4014806; doi:10.1186/1465-9921-14-100)
Supplement: Additional file 1 — RCT study and patient characteristics, individual study results, flow diagram, and NMA results at 12 weeks. Table S1. Key study characteristics for RCTs included in the network meta-analysis. Table S2. Individual study results for trough FEV1 at 12 weeks and 6 months (mL): difference in change from baseline (CFB) for treatment versus comparator. Table S3. Individual study results for post-dose FEV1 at 12 weeks and 6 months (mL): difference in change from baseline (CFB) for treatment versus comparator. Table S4. Individual study results for SGRQ total score at 12 weeks and 6 months: difference in change from baseline (CFB) for treatment versus comparator. Table S5. Individual study results for SGRQ responders at 12 weeks and 6 months: n/N (proportion responders) for each treatment. Table S6. Individual study results for TDI total score at 12 weeks and 6 months: difference in change from baseline (CFB) for treatment versus comparator. Table S7. Individual study results for TDI responders at 12 weeks and 6 months: n/N (proportion responders) for each treatment. Table S8. Results of base case network meta-analysis: Probability of each treatment being the best in terms of trough and post-dose FEV1 (mL), SGRQ total score and response, and TDI total score and response at 6 months. Figure S1. Flow diagram of study selection. Figure S2. Trough and post-dose FEV1 network meta-analysis results at 12 weeks: Difference in change from baseline (CFB) versus placebo. Figure S3. SGRQ total score network meta-analysis results at 6 months: Difference in change from baseline (CFB) or odds ratio (OR) versus placebo. Figure S4. TDI total score network meta-analysis results at 6 months: Difference in change from baseline (CFB) or odds ratio (OR) versus placebo. [file 1465-9921-14-100-S1.doc]

**Additional file 1**

**Table S1**. Key study characteristics for RCTs included in the network meta-analysis

| **Source** | **Design**[[1]](#footnote-2) | **[Centres/ Countries](../../../../C:%5CDocuments%20and%20Settings%5Cshalini.naik%5CLocal%20Settings%5CTemporary%20Internet%20Files%5CContent.MSO%5CD4E7867E.xls#RANGE!C90)**[[2]](#footnote-3) | **Treatment** | **Concomitant corticosteroids** | **Concomitant LABAs or LAMAs** | **Inclusion criteria**[[3]](#footnote-4) |
| --- | --- | --- | --- | --- | --- | --- |
| D’Urzu, 2011 (A2303) GLOW1 | 52 week RCT, PC, DB (except for TIO arm), MC | Multinational/ 200 centres | Tiotropium 18μg OD (n=268) vs.  Glycopyrronium OD (n=529) vs.  Placebo (n=269) | Yes (inhaled) | No (salbutamol/ albuterol as rescue) | Post-bronchodilator FEV_1_ ≥30% and <80%; post-bronchodilator FEV_1_/FVC < 70% |
| Kerwin, 2012 (A2304) GLOW2 | 26 week RCT, DB, PC, MC | Multinational/ 125 centres | Glycopyrronium OD (n=522) vs.  Placebo (n=270) | Yes (inhaled) | No (salbutamol/ albuterol as rescue) | Post-bronchodilator FEV_1_ ≥30% and <80%; post-bronchodilator FEV_1_/FVC < 70% |
| Kinoshita, 2012 (B1302) | 12 week RCT, PC, DB, MC | 73 centres; Japan, Taiwan, Korea, India, Hong Kong, and Singapore | Indacaterol 150μg OD (n=114) vs. Indacaterol 300μg OD (n=116) vs.  Placebo (n=117) | Yes (inhaled) | No (salbutamol as rescue) | Post bronchodilator FEV_1_ ≥30% and <80% of predicted normal value, post-bronchodilator FEV_1_/FVC<70%,excluded if exacerbation in 6 weeks prior |
| B2333 (NCT00792805) | 26 week RCT, PC, DB, MC | Multiple centres/  China and India | Indacaterol 150μg OD (n=187) vs. Indacaterol 300μg OD (n=188) vs.  Placebo (n=186) | Yes (inhaled) | No (salbutamol/ albuterol as rescue, slow-release theophylline) | Post bronchodilator FEV_1_ ≥30% and <80%; FEV_1_/FVC<70%; excluded if exacerbation in 6 weeks prior |
| Korn, 2011 (B2349) | 12 week RCT, DB, MC, DD | 142 centres/  8 countries | Indacaterol 150μg OD (n=560) vs. Salmeterol 50μg BID (n=1123) | Yes (inhaled) | No (salbutamol/ albuterol as rescue) | Post-bronchodilator FEV_1_ ≥30% and <80%, FEV_1_/FVC <70%; excluded if exacerbation in 6 weeks prior |
| Buhl, 2011 (B2350) | 12 week RCT, DB, DD, MC | NR | Indacaterol 150μg OD (n= 797) vs. Tiotropium 18μg OD (n=801) | Yes (inhaled) | No (salbutamol/ albuterol as rescue) | Post-bronchodilator FEV_1_ ≥30% and <80%; post-bronchodilator FEV_1_/FVC <70% |
| Kerwin, 2011 (B2354) | 12 week RCT, PC, DB, MC | # centres NR/USA | Indacaterol 75μg OD (n=163) vs. Placebo (n=160) | Yes (inhaled) | No (salbutamol/ albuterol as rescue) | FEV_1_ ≥30% and <80%; FEV_1_/FVC<70%; excluded if exacerbation in 6 weeks prior |
| Kerwin, 2011 (B2355) | 12 week RCT, PC, DB, MC | # centres NR/ USA | Indacaterol 75μg OD (n=159) vs. Placebo (n=159) | Yes (inhaled) | No (albuterol as rescue) | FEV_1_ ≥30% and <80%; FEV_1_/FVC<70%; excluded if exacerbation in 6 weeks prior |
| Jones, 2011  ACCLAIM COPD I | 52 week RCT,PC,DB | 139 centres in 16 European countries | Aclidinium 200μg OD (n=627) vs. Placebo (n=216) | Yes (inhaled, oral) | No (only salbutamol, oral sustained-release theophyllines) | FEV_1_ <80% of the predicted value; FEV_1_/FVC ratio of ≤70%; pre-dose FEV_1_ at randomization had to be within 80-120% of the pre bronchodilator FEV_1_ at screening |
| ACCLAIM  COPD II | 52 week RCT,PC,DB | 119 sites in 7 countries | Aclidinium 200μg OD (n=600) vs. Placebo (n=204) |
| Dahl, 2010 (B2334) | 52 week RCT, PC, DB, MC, DD | # centres NR/ 25 countries in S. American, Europe, Russia, Africa, and Asia | Indacaterol 300μg OD (n=437) vs. Formoterol 12μg BID (n=435) vs. Placebo (n=432) | Yes (inhaled) | No (salbutamol/ albuterol as rescue) | FEV_1_ ≥30% and <80%; FEV_1_/FVC<70%; reversible and non-reversible patients included; excluded if hospitalization 6 weeks prior to trial or during run-in period |
| Donohue, 2010 (B2335S) | 26 week RCT, PC, DB (except for tiotropium arm), MC, DD; Adaptive seamless | # centres NR/ Argentina, Canada, Europe, India, Italy, Korea, Taiwan, USA | Indacaterol 150μg OD (n=420) vs. Indacaterol 300μg OD (n=418) vs.  Tiotropium 18μg OD (n=420) vs.  Placebo (n=425) | Yes (inhaled) | No (salbutamol/ albuterol as rescue) | FEV_1_ ≥30% and <80%; FEV_1_/FVC<70%; reversible and non-reversible patients included; excluded if hospitalization 6 weeks prior |
| Kornmann, 2010 (B2336) | 26 week RCT, PC, DB, MC, DD | # centres NR/ Canada, Colombia, Europe and Russia, Slovakia, India, Peru, Taiwan | Indacaterol 150μg OD (n=333) vs. Salmeterol 50μg BID (n=334) vs. Placebo (n=335) | Yes (inhaled) | No (salbutamol/ albuterol as rescue) | FEV_1_ ≥30% and <80%; FEV_1_/FVC<70%; reversible and non-reversible patients included; excluded if hospitalization 6 weeks prior |
| Feldman, 2010 (B2346) | 12 week RCT, PC, DB, MC, DD | 103 centres NR/ USA, Australia/New Zealand, Belgium | Indacaterol 150μg OD (n=211) vs. Placebo (n=205) | Yes (inhaled) | No (salbutamol/ albuterol as rescue) | FEV_1_ ≥30% and <80%; FEV_1_/FVC<70%; reversible and non-reversible patients included; excluded if hospitalization 6 weeks prior |
| Bateman, 2010a | 48 week RCT, PC, DB, MC | 336 outpatient centres spanning 5 continents and involving 31 countries | Tiotropium 5μg OD (n=1989) vs.  Placebo (n=2002) | Yes (inhaled, oral; all respiratory medications permitted) | Yes LABAs (also salbutamol and all respiratory medications permitted) | Pre-bronchodilator FEV_1_ of ≤60% of predicted normal and FEV_1_/FVC of ≤70% |
| Bateman,  2010b | 48 week RCT, PC, DB, MC | NR | Tiotropium 5μg OD (n=670) vs.  Placebo(n=653) | Yes (inhaled, oral) | No (only theophylline, mucolytic agents, antileukotrines, salbutamol) | Pre-bronchodilator FEV_1_ ≤60% predicted and FEV_1_ ≤70% of FVC with a smoking history of ≥10 pack years |
| Gross, 2008 | 12 week RCT, PC, DB, DD, MC | 38 centres/ USA | Formoterol 12μg BID (n=114) vs.  Placebo (n=114) | Yes (inhaled, oral) | No | Post-bronchodilator FEV_1_ >30% and <70%; FEV_1_/FVC<70%; symptom criteria; excluded if exacerbation in 4 weeks prior |
| Moita, 2008 | 12 week RCT, PC, DB, MC | 31 centres/ Portugal | Tiotropium 18μg OD (n=147) vs.  Placebo (n=164) | Yes (inhaled, oral) | Yes LABAs (also theophylline except for 24 h preparations) | FEV_1_ ≤70%; FEV_1_/FVC ≤70%; excluded if ≥3 exacerbations previous year |
| Tashkin, 2008 (UPLIFT) | 4 year RCT, PC, DB, MC | 490 centres/ 37 countries | Tiotropium 18μg OD (n= 2987) vs.  Placebo (n=3006) | Yes (all respiratory medications except inhaled anticholinergics) | Yes LABAs (all respiratory medications except inhaled anticholinergics) | Post-bronchodilator FEV_1_ ≤70%; FEV_1_/FVC ≤70%; excluded if exacerbation 4 weeks prior |
| Tonnel, 2008 | 36 week RCT, PC, DB, MC | 123 centres/ France | Tiotropium 18μg OD (n=266) vs. Placebo (n=288) | Yes (inhaled, oral) | No (only theophylline except 24 hour preparations, mycolytics) | Pre- and post-bronchodilator FEV_1_ 20-70%; FEV_1_/FVC ≤ 70%; |
| Vogelmeier, 2008 | 24 week RCT, PC, DB (except for tiotropium arm),  MC | 86 centres in Germany, Italy, Netherlands, Russian Federation, Poland, Czech Republic, Spain and Hungary | Tiotropium 18μg OD (n=221) vs.  Placebo (n=209) | Yes (inhaled) | No (salbutamol) | FEV_1_ <70%, FEV_1_/FVC <70%; excluded if hospitalized due to acute exacerbation with prior month |
| Voshaar, 2008 | 12 week RCT,PC, DB, MC | 39 centres Germany, Italy, S. Africa, Switzerland and 25 centres USA and Canada | Tiotropium 5μg OD (n=180) vs.  Placebo (n=181) | Yes (inhaled, oral) | No (only theophylline, mucolytics) | Pre-bronchodilator FEV_1_ ≤ 60% of predicted normal, FEV_1_/FVC ≤ 70% , smoking history of ≥ 10 pack-years |
| Calverley, 2007 (TORCH) | 3 year RCT, PC, DB, MC | 444 centres/ 42 countries | Salmeterol 50μg BID (n=1542) vs. Placebo (n=1545) | No (usual medication except corticosteroids) | No (usual medication except LABAs/LAMAs) | Pre-bronchodilator FEV_1_ <60%; FEV_1_/FVC<70 %; <10% increase FEV_1_ predicted post-bronchodilator; |
| Chan, 2007 | 48 week RCT, PC, DB, MC | 101 centres/ Canada | Tiotropium 18μg OD (n=608) vs. Placebo (n=305) | Yes (inhaled, oral) | Yes LABAs (also theophylline, mucolytic preparations) | FEV_1_ ≤65%; FEV_1_/FVC ≤ 70%; included if ≥ 1 exacerbation previous year but not in 6 weeks prior |
| Verkindre, 2006 | 12 week RCT, PC, DB, MC | 10 centres/ France | Tiotropium 18μg OD (n=46) vs. Placebo (n=54) | Yes (inhaled, oral) | No (only theophylline, mucolytic agents) | FEV_1_ ≤50%; FEV_1_/SVC≤ 70%; residual volume ≥ 125%; excluded if unstable doses oral corticosteroid 6 weeks prior |
| Briggs, 2005 | 12 week RCT, DB, MC | 50 centres/ Europe, UK and USA | Tiotropium 18μg OD (n=328) vs. Salmeterol 50μg BID (n=325) | Yes (inhaled) | No (only usual medications different than LABAs) | FEV_1_ ≤60%; FEV_1_/FVC ≤70%; excluded if exacerbation 4 weeks prior |
| Covelli, 2005 | 12 week RCT, PC, DB, MC | 12 centres/ USA | Tiotropium 18μg OD (n=100) vs.  Placebo (n=96) | Yes (inhaled) | Yes LABAs (also theophyllines) | FEV_1_ ≤60%; FEV_1_/FVC ≤70%; excluded if exacerbation in prior 6 weeks |
| Niewoehner, 2005 | 24 week RCT, PC, DB, MC | 26 centres/ USA | Tiotropium 18μg OD (n=914) vs. Placebo (n=915) | Yes (inhaled, oral; all usual respiratory medications) | Yes LABAs (all other respiratory medications except anticholinergics) | FEV_1_ ≤60%; FEV_1_/FVC ≤70%; excluded if not recovered from exacerbation ≥ 30 days prior |
| Brusasco, 2003 | 24 week RCT, PC, DB, MC, DD | 18 countries | Tiotropium 18μg OD (n=402) vs. Salmeterol 50μg BID (405) vs.  Placebo (n=400) | NR | No (only theophylline) | FEV_1_ ≤65%; FEV_1_/FVC ≤ 70%; |
| Calverley, 2003 | 52 week RCT, PC, DB, MC | 196 centres/ 25 countries | Salmeterol 50μg BID (n=372) vs.  Placebo (n=361) | No | Yes LAMAs (also salbutamol, theophylline, mucolytics) | FEV_1_ 25-70%; FEV_1_ /FVC<70%; increase ≥ 10% FEV_1_ post bronchodilator.; Included if ≥1 exacerbation previous year and ≥1 exacerbation per year in previous 3 years |
| Celli, 2003 | 12 week RCT, PC, DB, MC, DD | 189 centres/ 15 countries | Salmeterol 50μg BID (n=554) vs. Placebo (n=271) | No | No (only usual medications other than β2-adrenoceptor agonists, anticholinergics, antibiotics, leukotriene antagonists) | FEV_1_ 20-70%;FEV_1_/FVC<65%;<15% reversibility FEV1;symptom criteria; excluded if exacerbation 6 weeks prior |
| Hanania, 2003 | 24 week RCT, PC, DB, MC | 76 centres/ USA | Salmeterol 50μg BID (n=177) vs. Placebo (n=185) | No | No (only theophylline) | FEV_1_ >40% and <65%; FEV_1_/FVC<70%; symptoms criteria; excluded if oral corticosteroids 6 weeks prior |
| Casaburi, 2002 | Two 56 week RCTs, PC, DB, MC | 50 centres/ countries NR | Tiotropium 18μg OD (n=550) vs. Placebo (n=371) | Yes (inhaled, oral) | No (only theophylline) | FEV_1_ ≤65%; FEV_1_/FVC ≤70%; |
| Chapman, 2002 | 24 week RCT, PC, DB, MC | 52 centres/ Canada, UK, Netherlands, Sweden, Russia Denmark | Salmeterol 50μg BID (n=201) vs. Placebo (n=207) | Yes (inhaled, oral) | Yes LAMAs (salbutamol, usual medications except beta-2-agonists) | FEV_1_ ≤85%; FEV_1_/FVC ≤70%; symptoms criteria; 5-15% reversibility FEV_1_ predicted; excluded if exacerbation 4 weeks prior |
| Donohue, 2002 | 36 week RCT, PC, DB, MC, DD | 39 countries/ 12 countries | Tiotropium 18μg OD (n=209) vs. Salmeterol 50μg BID (n=213) vs. Placebo (n=201) | Yes (inhaled, oral) | No (only theophylline) | FEV_1_ ≤60%; FEV_1_/FVC ≤70%; |
| Mahler, 2002 | 24 week RCT, PC, DB, MC, DD | 65 centres | Salmeterol 50μg BID (n=160) vs. Placebo (n=181) | No | No (only theophylline) | FEV_1_ <65% but >70L; FEV_1_/FVC ≤70%; excluded if moderate or severe exacerbation during run-in |
| Rossi, 2002 | 12 month RCT, PC,DB,MC | 81 centres worldwide | Formoterol 12μg BID (n=211) vs. Placebo (n=220) | Yes (inhaled, oral) | No (only salbutamol or albuterol) | FEV_1_<70% of the predicted value and ≥0.75 L, FEV_1_ vital capacity ratio of <88% of that predicted in men and <89% in women |
| Dahl, 2001 | 12 week RCT, PC, DB, DD, MC | 57 centres/ Europe, Russia, Canada, USA | Formoterol 12μg BID (n=194) vs. Placebo (n=200) | Yes (inhaled, oral) | No (only antibiotics) | FEV_1_ <70%; FEV_1_/FVC<88% for men and <89% for women; symptom criteria; excluded if used oral corticosteroids 4 weeks prior |
| Casaburi, 2000 | 13 week RCT, PC, DB, MC | 25 centres/ USA | Tiotropium; 18μg OD (279) vs. Placebo (n=191) | Yes (inhaled, oral) | No (only theophylline) | FEV_1_ ≤65%; FEV_1_/FVC ≤70% |
| Van Rutten, 1999 | 12 week RCT, PC, DB, MC , DD | 3 centres/ Netherlands | Salmeterol 50μg BID (n=47) vs. Placebo (n=50) | Yes (inhaled, oral; all maintenance drugs other than study drugs) | No (only maintenance drugs other than study drugs) | Pre-bronchodilator FEV_1_ 40-65%; FEV_1_/FVC ≤ 60% (post salbutamol); symptom criteria |

**Table S2. Individual study results for trough FEV_1_ at 12 weeks and 6 months (mL): Difference in change from baseline (CFB)** for treatment versus comparator

| Author, year | Placebo | TIO 18 | | SAL 50 | | FOR 12 | | TIO 5 | | ACL 200 | | IND 75 | | IND 150 | | IND 300 | | GPM 50 | |
| --- | --- | --- | --- | --- | --- | --- | --- | --- | --- | --- | --- | --- | --- | --- | --- | --- | --- | --- | --- |
|  |  | 12w | 6m | 12w | 6m | 12w | 6m | 12w | 6m | 12w | 6m | 12w | 6m | 12w | 6m | 12w | 6m | 12w | 6m |
| COVELLI, 2005* | C | 184  (37) |  |  |  |  |  |  |  |  |  |  |  |  |  |  |  |  |  |
| CHAN, 2007*† | C | 100  (14) |  |  |  |  |  |  |  |  |  |  |  |  |  |  |  |  |  |
| VERKINDRE, 2006 | C | 110  (40) |  |  |  |  |  |  |  |  |  |  |  |  |  |  |  |  |  |
| CASABURI, 2000 | C | 150  (14) |  |  |  |  |  |  |  |  |  |  |  |  |  |  |  |  |  |
| CASABURI, 2002 | C | 132  (14) | 153  (15) |  |  |  |  |  |  |  |  |  |  |  |  |  |  |  |  |
| MOITA, 2008* | C | 102  (31) |  |  |  |  |  |  |  |  |  |  |  |  |  |  |  |  |  |
| NIEWOEHNER, 2005* | C | 100  (10) | 100  (13) |  |  |  |  |  |  |  |  |  |  |  |  |  |  |  |  |
| TASHKIN, 2008* | C |  | 100  (6) |  |  |  |  |  |  |  |  |  |  |  |  |  |  |  |  |
| HANANIA, 2003 | C |  |  | 96  (27) | 92  (23) |  |  |  |  |  |  |  |  |  |  |  |  |  |  |
| MAHLER 2002 | C |  |  | 125  (22) | 92  (30) |  |  |  |  |  |  |  |  |  |  |  |  |  |  |
| CALVERLEY, 2003*† | C |  |  |  | 86  (16) |  |  |  |  |  |  |  |  |  |  |  |  |  |  |
| CHAPMAN, 2002* | C |  |  |  | 30  (21) |  |  |  |  |  |  |  |  |  |  |  |  |  |  |
| DONOHUE, 2002 | C |  | 137  (20) |  | 85  (20) |  |  |  |  |  |  |  |  |  |  |  |  |  |  |
| BRUSASCO, 2003 | C |  |  |  | 120  (16) |  | 90  (16) |  |  |  |  |  |  |  |  |  |  |  |  |
| GROSS, 2008 | C |  |  |  |  | 80  (27) |  |  |  |  |  |  |  |  |  |  |  |  |  |
| ROSSI 2002 | C |  |  |  |  | 40  (19) |  |  |  |  |  |  |  |  |  |  |  |  |  |
| VOSHAAR 2008 | C |  |  |  |  |  |  | 118  (23) |  |  |  |  |  |  |  |  |  |  |  |
| BATEMAN 2010a | C |  |  |  |  |  |  |  | 103  (8) |  |  |  |  |  |  |  |  |  |  |
| BATEMAN 2010b* | C |  |  |  |  |  |  |  | 124  (13) |  |  |  |  |  |  |  |  |  |  |
| BRIGGS, 2005 |  | 18  (15) |  | C |  |  |  |  |  |  |  |  |  |  |  |  |  |  |  |
| JONES, 2011a | C |  |  |  |  |  |  |  |  | 61  (14) |  |  |  |  |  |  |  |  |  |
| JONES, 2011b | C |  |  |  |  |  |  |  |  | 63  (14) |  |  |  |  |  |  |  |  |  |
| KERWIN, 2011 (55) | C |  |  |  |  |  |  |  |  |  |  | 140  (21) |  |  |  |  |  |  |  |
| KERWIN, 2011 (54) | C |  |  |  |  |  |  |  |  |  |  | 120  (19) |  |  |  |  |  |  |  |
| FELDMAN 2010 (46) | C |  |  |  |  |  |  |  |  |  |  |  |  | 130  (24) |  |  |  |  |  |
| BUHL, 2011 (50) |  | C |  |  |  |  |  |  |  |  |  |  |  | 0  (10) |  |  |  |  |  |
| KORN. 2011 (49) |  |  |  | C |  |  |  |  |  |  |  |  |  | 60  (11) |  |  |  |  |  |
| KORNMANN, 2011 (36) | C |  |  | 110  (18) | 110  (20) |  |  |  |  |  |  |  |  | 170  (18) | 180  (20) |  |  |  |  |
| DAHL, 2010 (34) | C |  |  |  |  | 70  (16) | 60  (19) |  |  |  |  |  |  |  |  | 170  (16) | 160  (19) |  |  |
| B2333 | C |  |  |  |  |  |  |  |  |  |  |  |  | 150  (20) | 140  (20) | 130  (20) | 140  (21) |  |  |
| KINOSHITA, 2012 (B1302) | C |  |  |  |  |  |  |  |  |  |  |  |  | 170  (20) |  | 200  (20) |  |  |  |
| DONOHUE, 2010 (35) | C | 140  (16) | 140  (18) |  |  |  |  |  |  |  |  |  |  | 180  (16) | 160  (19) | 180  (16) | 180 (18) |  |  |
| D’URZU, 2011 (A2304) | C |  |  |  |  |  |  |  |  |  |  |  |  |  |  |  |  | 108  (15) | 113  (17) |
| KERWIN, 2012 (A2303) | C | 83  (19) | 84  (22) |  |  |  |  |  |  |  |  |  |  |  |  |  |  | 97  (17) | 134  (19) |

*C=Comparator;*indicates LABA or LAMA use was permitted during trial; †indicates patients included in the trial had an exacerbation history.*

**Table S3. Individual study results for post-dose FEV_1_ at 12 weeks and 6 months (mL): Difference in change from baseline (CFB)** for treatment versus comparator

| Author, year | Placebo | TIO 18 | | SAL 50 | | FOR 12 | | TIO 5 | | IND 75 | | IND 150 | | IND 300 | | GPM 50 | |
| --- | --- | --- | --- | --- | --- | --- | --- | --- | --- | --- | --- | --- | --- | --- | --- | --- | --- |
|  |  | 12w | 6m | 12w | 6m | 12w | 6m | 12w | 6m | 12w | 6m | 12w | 6m | 12w | 6m | 12w | 6m |
| CASABURI, 2000 | C | 228  (13) |  |  |  |  |  |  |  |  |  |  |  |  |  |  |  |
| CASABURI, 2002 | C | 210  (14) | 232  (18) |  |  |  |  |  |  |  |  |  |  |  |  |  |  |
| VOGELMEIER, 2009 | C |  | 170  (42) |  |  |  |  |  |  |  |  |  |  |  |  |  |  |
| HANANIA, 2003 | C |  |  | 153  (27) | 140  (32) |  |  |  |  |  |  |  |  |  |  |  |  |
| MAHLER 2002 | C |  |  | 180  (25) | 191  (27) |  |  |  |  |  |  |  |  |  |  |  |  |
| DONOHUE, 2002 | C |  | 240  (27) |  | 160  (27) |  |  |  |  |  |  |  |  |  |  |  |  |
| BRUSASCO, 2003 | C |  | 220  (19) |  | 150  (19) |  |  |  |  |  |  |  |  |  |  |  |  |
| DAHL, 2001 | C |  |  |  |  | 340  (21) |  |  |  |  |  |  |  |  |  |  |  |
| GROSS, 2008 | C |  |  |  |  | 270  (28) |  |  |  |  |  |  |  |  |  |  |  |
| ROSSI 2002 | C |  |  |  |  | 210  (20) |  |  |  |  |  |  |  |  |  |  |  |
| VOSHAAR 2008 | C |  |  |  |  |  |  | 194  (23) |  |  |  |  |  |  |  |  |  |
| BRIGGS, 2005 |  | C |  | -50  (14) |  |  |  |  |  |  |  |  |  |  |  |  |  |
| KERWIN, 2011 (55) | C |  |  |  |  |  |  |  |  | 190  (26) |  |  |  |  |  |  |  |
| KERWIN, 2011 (54) | C |  |  |  |  |  |  |  |  | 170  (20) |  |  |  |  |  |  |  |
| FELDMAN 2010 (46) | C |  |  |  |  |  |  |  |  |  |  | 170  (27) |  |  |  |  |  |
| BUHL, 2011 (50) |  | C |  |  |  |  |  |  |  |  |  | -20  (10) |  |  |  |  |  |
| KORN. 2011 (49) |  |  |  | C |  |  |  |  |  |  |  | 50  (13) |  |  |  |  |  |
| KORNMANN, 2011 (36) | C |  |  | 200  (34) | 210  (37) |  |  |  |  |  |  | 190  (33) | 240  (36) |  |  |  |  |
| DAHL, 2010 (34) | C |  |  |  |  | 190  (33) |  |  |  |  |  |  |  | 250  (33) |  |  |  |
| KINOSHITA, 2012 (B1302) | C |  |  |  |  |  |  |  |  |  |  | 230  (32) |  | 250  (32) |  |  |  |
| DONOHUE, 2010 (35) | C | 180  (34) | 190  (37) |  |  |  |  |  |  |  |  | 230  (35) | 220  (38) | 230  (34) | 270  (37) |  |  |
| D’URZU, 2011 (A2304) | C |  |  |  |  |  |  |  |  |  |  |  |  |  |  | 192  (17) | 209  (19) |
| KERWIN, 2012 (A2303) | C | 136  (21) | 136  (23) |  |  |  |  |  |  |  |  |  |  |  |  | 172  (19) | 182  (20) |

*C=Comparator;*indicates LABA or LAMA use was permitted during trial; †indicates patients included in the trial had an exacerbation history.*

**Table S4. Individual study results for SGRQ total score at 12 weeks and 6 months: Difference in change from baseline (CFB) for treatment versus comparator**

| Author, year | Placebo | TIO 18 | | SAL 50 | | FOR 12 | | TIO 5 | | IND 75 | | IND 150 | | IND 300 | | GPM 50 | |
| --- | --- | --- | --- | --- | --- | --- | --- | --- | --- | --- | --- | --- | --- | --- | --- | --- | --- |
|  |  | 12w | 6m | 12w | 6m | 12w | 6m | 12w | 6m | 12w | 6m | 12w | 6m | 12w | 6m | 12w | 6m |
| VERKINDRE, 2006 | C | -6.50  (2.90) |  |  |  |  |  |  |  |  |  |  |  |  |  |  |  |
| TONNEL, 2008 | C | -3.47  (1.10) | -3.50  (1.24) |  |  |  |  |  |  |  |  |  |  |  |  |  |  |
| CELLI, 2003 | C |  |  | -2.10  (1.28) |  |  |  |  |  |  |  |  |  |  |  |  |  |
| CASABURI, 2002 | C |  | -3.10  (0.88) |  |  |  |  |  |  |  |  |  |  |  |  |  |  |
| TASHKIN, 2008* | C |  | -2.90  (0.42) |  |  |  |  |  |  |  |  |  |  |  |  |  |  |
| VOGELMEIER, 2008 | C |  | -2.09  (1.27) |  |  |  |  |  |  |  |  |  |  |  |  |  |  |
| CALVERLEY, 2003*† | C |  |  |  | -1.41  (0.97) |  |  |  |  |  |  |  |  |  |  |  |  |
| CALVERLEY, 2007 | C |  |  |  | -0.35  (0.58) |  |  |  |  |  |  |  |  |  |  |  |  |
| CHAPMAN, 2002* | C |  |  |  | -1.50  (1.44) |  |  |  |  |  |  |  |  |  |  |  |  |
| DONOHUE, 2002 | C |  | -2.71  (1.42) |  | -1.11  (1.42) |  |  |  |  |  |  |  |  |  |  |  |  |
| BRUSASCO, 2003 | C |  | -2.70  (0.99) |  | -1.30  (0.99) |  |  |  |  |  |  |  |  |  |  |  |  |
| GROSS, 2008 | C |  |  |  |  | -3.51  (1.63) |  |  |  |  |  |  |  |  |  |  |  |
| DAHL, 2001 | C |  |  |  |  | -5.10  (1.83) |  |  |  |  |  |  |  |  |  |  |  |
| BATEMAN 2010a | C |  |  |  |  |  |  |  | -2.20  (0.46) |  |  |  |  |  |  |  |  |
| KERWIN, 2011 (55) | C |  |  |  |  |  |  |  |  | -3.60  (1.40) |  |  |  |  |  |  |  |
| KERWIN, 2011 (54) | C |  |  |  |  |  |  |  |  | -3.80  (1.21) |  |  |  |  |  |  |  |
| FELDMAN 2010 (46) | C |  |  |  |  |  |  |  |  |  |  | -4.75  (1.22) |  |  |  |  |  |
| BUHL, 2011 (50) |  | C |  |  |  |  |  |  |  |  |  | -2.10  (0.57) |  |  |  |  |  |
| KORNMANN, 2011 (36) | C |  |  | -4.20  (1.01) | -4.00  (1.12) |  |  |  |  |  |  | -6.30  (0.99) | -5.24  (1.19) |  |  |  |  |
| DAHL, 2010 (34) | C |  |  |  |  | -3.20  (0.90) | -3.00  (0.96) |  |  |  |  |  |  | -3.80  (0.90) | -4.40  (0.95) |  |  |
| B2333 | C |  |  |  |  |  |  |  |  |  |  | -2.70  (1.40) | -2.30  (1.57) | -3.10  (1.40) | -2.60  (1.58) |  |  |
| KINOSHITA, 2012 (B1302) | C |  |  |  |  |  |  |  |  |  |  | -4.80  (1.72) |  | -5.70  (1.75) |  |  |  |
| DONOHUE, 2010 (35) | C | -1.10  (0.86) | -1.00  (0.92) |  |  |  |  |  |  |  |  | -2.80  (0.87) | -3.30  (0.92) | -2.50  (0.86) | -2.40  (0.91) |  |  |
| D’URZU, 2011 (A2304) | C |  |  |  |  |  |  |  |  |  |  |  |  |  |  | -2.71  (0.86) | -2.81  (0.96) |
| KERWIN, 2012 (A2303) | C | -2.84  (0.97) | -2.52  (1.11) |  |  |  |  |  |  |  |  |  |  |  |  | -3.17  (0.84) | -3.38  (0.97) |

*C=Comparator;*indicates LABA or LAMA use was permitted during trial; †indicates patients included in the trial had an exacerbation history.*

**Table S5. Individual study results for SGRQ responders at 12 weeks and 6 months: n/N (proportion responders) for each** treatment

| Author, year | Placebo | | TIO 18 | | SAL 50 | | FOR 12 | | TIO 5 | | ACL 200 | | IND 75 | | IND 150 | | IND 300 | | GPM 50 | |
| --- | --- | --- | --- | --- | --- | --- | --- | --- | --- | --- | --- | --- | --- | --- | --- | --- | --- | --- | --- | --- |
|  | 12w | 6m | 12w | 6m | 12w | 6m | 12w | 6m | 12w | 6m | 12w | 6m | 12w | 6m | 12w | 6m | 12w | 6m | 12w | 6m |
| VERKINDRE, 2006 | 16/46  (35%) |  | 26/44  (59%) |  |  |  |  |  |  |  |  |  |  |  |  |  |  |  |  |  |
| TONNEL, 2008 | 110/245  (45%) |  | 147/247  (59%) |  |  |  |  |  |  |  |  |  |  |  |  |  |  |  |  |  |
| VAN RUTTEN, 1999 | 10/45  (23%) |  |  |  | 10/43  (24%) |  |  |  |  |  |  |  |  |  |  |  |  |  |  |  |
| DONOHUE, 2002 |  | 67/159  (42%) |  | 95/186  (51%) |  | 75/187  (40%) |  |  |  |  |  |  |  |  |  |  |  |  |  |  |
| BRUSASCO, 2003 |  | 157/400  (39%) |  | 197/402  (49%) |  | 175/405  (43%) |  |  |  |  |  |  |  |  |  |  |  |  |  |  |
| BATEMAN 2010a |  | 701/1953  (36%) |  |  |  |  |  |  |  | 860/1939  (44%) |  |  |  |  |  |  |  |  |  |  |
| JONES, 2011a | 77/216  (36%) |  |  |  |  |  |  |  |  |  | 278/627  (44%) |  |  |  |  |  |  |  |  |  |
| JONES, 2011b | 49/204  (24%) |  |  |  |  |  |  |  |  |  | 226/600  (38%) |  |  |  |  |  |  |  |  |  |
| KERWIN, 2011 (55) | 54/145  (37%) |  |  |  |  |  |  |  |  |  |  |  | 75/148  (51%) |  |  |  |  |  |  |  |
| KERWIN, 2011 (54) | 49/142  (35%) |  |  |  |  |  |  |  |  |  |  |  | 70/147  (48%) |  |  |  |  |  |  |  |
| FELDMAN 2010 (46) | 62/187  (33%) |  |  |  |  |  |  |  |  |  |  |  |  |  | 104/199  (52%) |  |  |  |  |  |
| BUHL, 2011 (50) |  |  | 320/753  (43%) |  |  |  |  |  |  |  |  |  |  |  | 375/743  (51%) |  |  |  |  |  |
| KORNMANN, 2011 (36) | 115/294  (39%) | 104/274  (38%) |  |  | 141/301  (47%) | 142/292  (49%) |  |  |  |  |  |  |  |  | 179/309  (58%) | 158/299  (53%) |  |  |  |  |
| DAHL, 2010 (34) | 143/347  (41%) | 118/294  (40%) |  |  |  |  | 185/359  (52%) | 162/318  (51%) |  |  |  |  |  |  |  |  | 193/372  (52%) | 181/330  (55%) |  |  |
| B2333 | 98/164  (60%) | 94/155  (61%) |  |  |  |  |  |  |  |  |  |  |  |  | 111/171  (65%) | 106/163  (65%) | 107/175  (61%) | 99/161  (61%) |  |  |
| KINOSHITA, 2012 (B1302) | 38/101  (38%) |  |  |  |  |  |  |  |  |  |  |  |  |  | 55/108  (51%) |  | 59/107  (55%) |  |  |  |
| DONOHUE, 2010 (35) | 156/347  (45%) | 146/319  (46%) | 168/374  (45%) | 169/357  (47%) |  |  |  |  |  |  |  |  |  |  | 191/368  (52%) | 200/346  (58%) | 188/375  (50%) | 189/360  (53%) |  |  |
| D’URZU, 2011 (A2304) | 117/246  (48%) | 114/246  (46%) |  |  |  |  |  |  |  |  |  |  |  |  |  |  |  |  | 283/497  (57%) | 285/502  (57%) |
| KERWIN, 2012 (A2303) | 112/247 (45%) | 121/248  (49%) | 146/249  (59%) | 153/251  (61%) |  |  |  |  |  |  |  |  |  |  |  |  |  |  | 287/496  (58%) | 294/499  (59%) |

*C=Comparator;*indicates LABA or LAMA use was permitted during trial; †indicates patients included in the trial had an exacerbation history.*

**Table S6. Individual study results for TDI total score at 12 weeks and 6 months: Difference in TDI for treatment versus comparator**

| Author, year | Placebo | TIO 18 | | SAL 50 | | FOR 12 | | IND 75 | | IND 150 | | IND 300 | | GPM 50 | |
| --- | --- | --- | --- | --- | --- | --- | --- | --- | --- | --- | --- | --- | --- | --- | --- |
|  |  | 12w | 6m | 12w | 6m | 12w | 6m | 12w | 6m | 12w | 6m | 12w | 6m | 12w | 6m |
| VERKINDRE, 2006 | C | 1.28  (0.89) |  |  |  |  |  |  |  |  |  |  |  |  |  |
| CASABURI, 2002 | C | 0.95  (0.20) | 0.85  (0.19) |  |  |  |  |  |  |  |  |  |  |  |  |
| HANANIA, 2003 | C |  |  |  | 0.70  (0.32) |  |  |  |  |  |  |  |  |  |  |
| MAHLER 2002 | C |  |  | 0.70  (0.31) | 0.50  (0.33) |  |  |  |  |  |  |  |  |  |  |
| DONOHUE, 2002 | C |  | 1.02  (0.33) |  | 0.24  (0.34) |  |  |  |  |  |  |  |  |  |  |
| BRUSASCO, 2003 | C |  | 1.10  (0.30) |  | 0.70  (0.30) |  |  |  |  |  |  |  |  |  |  |
| KERWIN, 2011 (55) | C |  |  |  |  |  |  | 0.45  (0.33) |  |  |  |  |  |  |  |
| KERWIN, 2011 (54) | C |  |  |  |  |  |  | 1.23  (0.34) |  |  |  |  |  |  |  |
| BUHL, 2011 (50) |  | C |  |  |  |  |  |  |  | 0.58  (0.15) |  |  |  |  |  |
| KORN. 2011 (49) | C |  |  |  |  |  |  |  |  | 0.63  (0.17) |  |  |  |  |  |
| KORNMANN, 2011 (36) | C |  |  | 0.90  (0.23) | 0.97  (0.25) |  |  |  |  | 1.45  (0.23) | 0.99  (0.24) |  |  |  |  |
| DAHL, 2010 (34) | C |  |  |  |  | 0.72  (0.21) | 0.69  (0.22) |  |  |  |  | 1.17  (0.21) | 1.32  (0.22) |  |  |
| B2333 | C |  |  |  |  |  |  |  |  | 0.76  (0.28) | 0.82  (0.28) | 0.73  (0.27) | 1.15  (0.27) |  |  |
| KINOSHITA, 2012 (B1302) | C |  |  |  |  |  |  |  |  | 1.30  (0.34) |  | 1.26  (0.35) |  |  |  |
| DONOHUE, 2010 (35) | C | 0.75  (0.22) | 0.87  (0.23) |  |  |  |  |  |  | 0.93  (0.22) | 1.00  (0.23) | 1.18  (0.22) | 1.18  (0.23) |  |  |
| D’URZU, 2011 (A2304) | C |  |  |  |  |  |  |  |  |  |  |  |  | 0.73  (0.23) | 1.04  (0.24) |
| KERWIN, 2012 (A2303) | C | 0.26  (0.30) | 0.94  (0.30) |  |  |  |  |  |  |  |  |  |  | 0.60  (0.27) | 0.81  (0.26) |

**indicates LABA or LAMA use was permitted during trial; †indicates patients included in the trial had an exacerbation history.*

**Table S7. Individual study results for TDI responders at 12 weeks and 6 months: n/N (proportion responders) for each** treatment

| Author, year | Placebo | | TIO 18 | | SAL 50 | | FOR 12 | | IND 75 | | IND 150 | | IND 300 | | GPM 50 | |
| --- | --- | --- | --- | --- | --- | --- | --- | --- | --- | --- | --- | --- | --- | --- | --- | --- |
|  | 12w | 6m | 12w | 6m | 12w | 6m | 12w | 6m | 12w | 6m | 12w | 6m | 12w | 6m | 12w | 6m |
| DONOHUE, 2002 |  | 38/148  (26%) |  | 77/184  (42%) |  | 63/179  (35%) |  |  |  |  |  |  |  |  |  |  |
| BRUSASCO, 2003 |  | 119/400  (30%) |  | 173/402  (43%) |  | 167/405  (41%) |  |  |  |  |  |  |  |  |  |  |
| KERWIN, 2011 (55) | 53/149  (36%) |  |  |  |  |  |  |  | 69/148  (47%) |  |  |  |  |  |  |  |
| KERWIN, 2011 (54) | 48/150  (32%) |  |  |  |  |  |  |  | 73/150  (49%) |  |  |  |  |  |  |  |
| BUHL, 2011 (50) |  |  | 369/737  (50%) |  |  |  |  |  |  |  | 422/729  (58%) |  |  |  |  |  |
| KORN. 2011 (49) |  |  |  |  | 328/523  (63%) |  |  |  |  |  | 353/509  (69%) |  |  |  |  |  |
| KORNMANN, 2011 (36) | 113/286  (40%) | 123/272  (45%) |  |  | 152/296  (51%) | 155/289  (54%) |  |  |  |  | 182/303  (60%) | 168/297  (57%) |  |  |  |  |
| DAHL, 2010 (34) | 137/343  (40%) | 116/284  (41%) |  |  |  |  | 190/359  (53%) | 171/316  (54%) |  |  |  |  | 229/364  (63%) | 193/325  (59%) |  |  |
| B2333 | 101/163  (62%) | 86/155  (55%) |  |  |  |  |  |  |  |  | 122/167  (73%) | 117/158  (74%) | 122/172  (71%) | 125/159  (79%) |  |  |
| KINOSHITA, 2012 (B1302) | 40/102  (39%) |  |  |  |  |  |  |  |  |  | 66/108  (61%) |  | 58/107  (54%) |  |  |  |
| DONOHUE, 2010 (35) | 138/326  (42%) | 144/309  (47%) | 198/360  (55%) | 200/349  (57%) |  |  |  |  |  |  | 209/355  (59%) | 214/343  (62%) | 239/363  (66%) | 250/353  (71%) |  |  |
| D’URZU, 2011 (A2304) | 114/236  (48%) | 116/240 (48%) |  |  |  |  |  |  |  |  |  |  |  |  | 281/484  (58%) | 302/493  (61%) |
| KERWIN, 2012 (A2303) | 95/209  (46%) | 96/217  (44%) | 121/232  (52%) | 127/238  (53%) |  |  |  |  |  |  |  |  |  |  | 262/459  (57%) | 260/470  (55%) |

**indicates LABA or LAMA use was permitted during trial; †indicates patients included in the trial had an exacerbation history.*

**Table S8. Results of base case network meta-analysis: Probability of each treatment being the best in terms of trough and post-dose FEV_1_ (mL), SGRQ total score and response, and TDI total score and response at** 6 months

| **Intervention** | **CFB in trough FEV_1_ vs. placebo** | **CFB in post-dose FEV_1_ vs. placebo** | **CFB in SGRQ total score vs. placebo** | **OR for SGRQ responders vs. placebo** | **TDI total score vs. placebo** | **OR for TDI responders vs. placebo** |
| --- | --- | --- | --- | --- | --- | --- |
| TIO 18 | 0% | 1% | 0% | 3% | 4% | 0% |
| SAL 50 | 0% | 1% | 0% | 0% | 0% | 0% |
| FOR 12 | 0% | NR | 6% | 13% | 0% | 4% |
| TIO 5 | 0% | NR | 1% | 11% | NR | NR |
| IND 150 | 29% | 10% | 52% | 51% | 2% | 1% |
| IND 300 | 64% | 83% | 25% | 10% | 86% | 95% |
| GPM 50 | 6% | 6% | 15% | 12% | 7% | 1% |

*FEV_1_=Forced expiratory volume; FOR 12= Formoterol 12µg twice daily (BID); GLM 50= glycopyrronium 50µg once daily (OD); IND 75= Indacaterol 75µg OD; IND 150= Indacaterol 150µg OD; IND 300= Indacaterol 300µg OD; NR= Not reported; SAL 50= Salmeterol 50µg BID; SGRQ= St. George’s Respiratory Questionnaire; TIO 5= Tiotropium 5µg OD; TIO 18= Tiotropium 18µg OD; TDI=Transitional Dyspnoea Index*

**Figure S1. Flow diagram of study selection**

600 Abstracts identified by OVID

173 Abstracts identified by Cochrane

References excluded: (436)

Trial design (124)

Patient population (24)

Intervention (90)

Comparators (38)

Trial duration ≤ 6 weeks (96)

Insufficient data [Conference abstracts] (56)

Repeat abstracts (189)

96 Full-text articles reviewed

References excluded: (54)

Intervention dose (15)

Trial design (16)

Patient population (1)

Intervention (3)

Outcomes (4)

Trial duration ≤ 6 weeks (13)

Repeat papers (2)

50 Full-text articles and 51 RCTs identified

[42 full text publications + 7 CSRs +1 aclidinium publication (2 RCTs)]

Additional data:

B2334 (Dahl 2010),

B2335S (Donohue 2010),

B2336 (Kornmann 2011),

B2346 (Feldman 2010),

B2349 (Korn 2011),

B2354 (Kerwin 2011),

B2355 (Kerwin 2011),

B1302 (Kinoshita 2012),

B2333 (NCT00792805),

B2350 (Buhl, 2011),

A2303 (Kerwin, 2012)

A2304 (D’Urzu, 2011),

Jones 2011 (Medline in

Process)

39 Full-text articles and 40 RCTs analyzed

[31 full text publications + 7 CSRs +1 aclidinium publication (2 RCTs)]

Full-text excluded from analysis: (5)

No outcomes reported <2 weeks from 12 week or 6 month time point

Full-text excluded from analysis: (6)

Only exacerbations outcomes reported

**Figure S2. Trough and post-dose FEV_1_ network meta-analysis results at 12 weeks: Difference in change from baseline (CFB) versus placebo**

*Bars represent 95% Credible Interval; CFB=Change from baseline; FEV_1_=Forced expiratory volume; FOR 12= Formoterol 12µg twice daily (BID); GPM 50= glycopyrronium 50µg once daily (OD); IND 75= Indacaterol 75µg OD; IND 150= Indacaterol 150µg OD; IND 300= Indacaterol 300µg OD; SAL 50= Salmeterol 50µg BID; TIO 5= Tiotropium 5µg OD; TIO 18= Tiotropium 18µg OD;*

**Figure S3. SGRQ network meta-analysis results at 6 months: Difference in change from baseline (CFB) or odds ratio (OR) versus placebo**

*Bars represent 95% Credible Interval; CFB=Change from baseline; FOR 12= Formoterol 12µg twice daily (BID); GPM 50= glycopyrronium 50µg once daily (OD); IND 75= Indacaterol 75µg OD; IND 150= Indacaterol 150µg OD; IND 300= Indacaterol 300µg OD; OR=Odds ratio; SAL 50= Salmeterol 50µg BID; SGRQ=St. George’s Respiratory Questionnaire; TIO 5= Tiotropium 5µg OD; TIO 18= Tiotropium 18µg OD;*

**Figure S4. TDI network meta-analysis results at 6 months: Difference in TDI or odds ratio (OR) versus placebo**

*Bars represent 95% Credible Interval; CFB=Change from baseline; FOR 12= Formoterol 12µg twice daily (BID); GPM 50= glycopyrronium 50µg once daily (OD); IND 75= Indacaterol 75µg OD; IND 150= Indacaterol 150µg OD; IND 300= Indacaterol 300µg OD; OR=Odds ratio; SAL 50= Salmeterol 50µg BID; TDI=Transition Dyspnoea Index; TIO 5= Tiotropium 5µg OD; TIO 18= Tiotropium 18µg OD;*

**Search Strategy for updating the systematic review from 2010-2011 in EMBASE and MEDLINE**

1 (formoterol or eformoterol or foradil or oxis or atimos modulite or atock or perforomist or salmeterol or serevent or tiotropium or spiriva or Ba 679 BR or Indacaterol or onbrez or arcapta or NVA-237 or NVA237 or (NVA adj "237") or glycopyrronium bromide or glycopyrrolate or aclidinium bromide).ab. or (formoterol or eformoterol or foradil or oxis or atimos modulite or atock or perforomist or salmeterol or serevent or tiotropium or spiriva or Ba 679 BR or Indacaterol or onbrez or arcapta or NVA-237 or NVA237 or (NVA adj "237") or glycopyrronium bromide or glycopyrrolate or aclidinium bromide).ti.

2 exp Pulmonary Disease, Chronic Obstructive/

3 (COPD or chronic obstructive pulmonary disease or COAD or chronic obstructive airway disease or chronic obstructive lung disease or chronic bronchitis or emphysema).ab. or (COPD or chronic obstructive pulmonary disease or COAD or chronic obstructive airway disease or chronic obstructive lung disease or chronic bronchitis or emphysema).ti.

4 2 or 3

5 (randomised or randomized or randomly or placebo or trial).ab. or (randomised or randomized or randomly or placebo or trial).ti.

6 exp RANDOMIZED CONTROLLED TRIAL/

7 exp controlled clinical trial/

8 5 or 6 or 7

9 1 and 4

10 8 and 9

11 limit 10 to english language

12 limit 11 to human

13 limit 12 to yr="2010 -Current"

14 limit 13 to humans

15 remove duplicates from 14

1. *RCT=randomized clinical trial; PC=placebo-controlled; DB=double-blind; MC=multi-centre; NR=not reported;*  [↑](#footnote-ref-2)
2. *UK=United Kingdom; USA=United Sates of America; S. America=South America;*  [↑](#footnote-ref-3)
3. *FEV_1_=forced expiratory volume in 1 second; FVC=Forced vital capacity; wks=weeks; ICS=inhaled corticosteroid; LABA=long-acting beta-agonist; h=hour;*  [↑](#footnote-ref-4)
